# Patterns of genetic variation and morphology support the recognition of five species in the *Gaultheria leucocarpa* Blume (Ericaceae) group from mainland China

**DOI:** 10.1002/ece3.10178

**Published:** 2023-06-09

**Authors:** Yi‐Rong Li, Yan‐Ling Xu, Peter W. Fritsch, Lu Lu

**Affiliations:** ^1^ School of Pharmaceutical Sciences and Yunnan Key Laboratory of Pharmacology for Natural Products Kunming Medical University Kunming Yunnan China; ^2^ Botanical Research Institute of Texas Fort Worth Texas USA

**Keywords:** diagnostic character, new species, phylogenetics, population genetics, taxonomic treatment

## Abstract

*Gaultheria leucocarpa* and its varieties form a clade of aromatic shrubs that is widely distributed in subtropical and East Asian tropical regions. The group is taxonomically difficult and in need of thorough taxonomic investigation. This study focused on taxonomic delimitation within the *G. leucocarpa* group from mainland China. Field surveys covering the distributional range of *G. leucocarpa* in mainland China were conducted, wherein four populations from Yunnan and one from Hunan were found bearing morphological and habitat differences. A 63‐species phylogenetic tree of *Gaultheria* based on one nuclear and three chloroplast markers that included samples from the *G. leucocarpa* group was reconstructed with maximum likelihood to clarify the monophyly of the *G. leucocarpa* group. Taxonomic relationships among populations were investigated with morphology and population genetics, the latter by using two chloroplast genes and two low‐copy nuclear genes. Based on the sum of morphological and genetic analyses, we described three species of *Gaultheria* as new to science, clarified the taxonomic status of *G. leucocarpa* var. *pingbienensis*, elevating it to the species level, and resurrected *G. crenulata* and treated the varieties *G. leucocarpa* var. *crenulata*, and *G. leucocarpa* var. *yunnanensis* as synonyms of this species. We provide a key to the five species now recognized, along with descriptions and photographs.

## Graphical abstract

By integrating morphology, DNA sequences, and multiple field surveys, we first clarify the taxonomy of the *Gaultheria leucocarpa* group from mainland China with elevation of all varieties to species in this study. This work has resulted in an increase of species within series *Gymnobotrys* of *Gaultheria* and will facilitate investigations on the medicinal value of this group of plants.

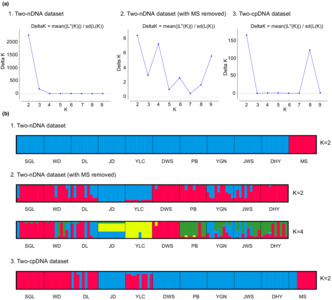

## 1 INTRODUCTION

*Gaultheria leucocarpa* Blume (also referred to herein as the “*G. leucocarpa* group”) belongs to the Gymnobotrys clade of the genus *Gaultheria* Kalm ex L. within the tribe Gaultherieae Nied. of Ericaceae Juss. (Fritsch et al., 2011; Lu et al., 2019), with a widespread distribution throughout subtropical and tropical eastern Asia within elevations of 200–3300 m (Fang & Stevens, 2005; Fritsch et al., 2008; Sleumer, 1957; YRL & LL, pers. obs.). The branches and leaves of this species are rich in aromatic oil, mainly methyl salicylate (Chua & Sunarti, 1999; Nikolić et al., 2013), used in traditional ethnic medicine (Liu et al., 2013; Ma et al., 2001; Mustaqim & Setiawan, 2020; Silalahi, 2018). *Gaultheria leucocarpa* displays considerable variation in morphology over its widespread geographic distribution (Copeland, 1932; Hsu, 1981; Sleumer, 1957). Reflecting this variation, it has been classified into six varieties (one with four forms; World Flora Online, http://www.worldfloraonline.org; Mustaqim & Setiawan, 2020; Sleumer, 1967). The varieties are mainly delimited by the color of mature fruits, leaves, inflorescence, and corolla, and whether the ovary is glabrous or pubescent.

*Gaultheria leucocarpa* var. *yunnanensis* (Franch.) T.Z. Xu & R.C. Fang is the most widespread variety within this species, occurring nearly throughout the area of China south of the Yangtze River Basin and Taiwan (Fang & Stevens, 2005). It is characterized by glabrous branchlets and leaves, filaments with various trichomes, a pubescent ovary, and deep bluish black fruits at maturity (Fang & Stevens, 2005; Table S1). Because of high morphological variation, its taxonomy has been long debated. Taxonomic revisions have either elevated it to the species level or reduced it to a variety of *G. leucocarpa*. Hsu (1981) recognized four varieties of this species in mainland China, that is, *G. leucocarpa* var. *hirsuta* (D. Fang & N.K. Liang) T.Z. Xu, *G. leucocarpa* var. *cumingiana* (S. Vidal) T.Z. Hsu, *G. leucocarpa* var. *crenulata* (Kurz) T.Z. Xu, and *G. leucocarpa* var. *pingbienensis* C.Y. Wu ex T.Z. Xu. In the *Flora of China* treatment of the Ericaceae, Fang and Stevens (2005) accepted only two mainland China varieties, that is, *G. leucocarpa* var. *yunnanensis* and *G. leucocarpa* var. *crenulata*. The latter occurs south of the Yangtze River Basin and was distinguished from *G. leucocarpa* var. *yunnanensis* only by its glandular‐hirsute twigs, petioles, leaf margins, and inflorescences (Fritsch et al., 2008). However, analysis of plastid data indicated that samples of *G. leucocarpa* var. *crenulata* nested within those of *G. leucocarpa* var. *yunnanensis*; the analysis did not support *G. leucocarpa* var. *crenulata* as a variety (Li et al., 2020).

Another variety in mainland China with taxonomic controversy is *G. leucocarpa* var. *pingbienensis*, collected from Pingbian County, Yunnan Province (barcode KUN 1208603). This variety was differentiated by coriaceous elliptical leaves but treated as a synonym of *G. leucocarpa* var. *yunnanensis* by Fang and Stevens (2005). Fritsch et al. (2008) placed *G. leucocarpa* var. *yunnanensis* in the synonymy of *G. leucocarpa* var. *pingbienensis* because they considered these to be the same variety and the varietal epithet *pingbienensis* has nomenclatural priority over the varietal epithet *yunnanensis*. In the description of Hsu (1981), no diagnostic characters for *G. leucocarpa* var. *pingbienensis* were mentioned except for what appears to be taxonomically trivial leaf morphology.

During 2017–2021, we conducted six field surveys wherein we collected 84 populations of *G. leucocarpa* throughout mainland China. In addition to representative populations of *G. leucocarpa* var. *yunnanensis*, *G. leucocarpa* var. *crenulata*, and *G. leucocarpa* var. *pingbienensis*, we found three more populations whose characters appeared not to match well those of the three named mainland varieties and also differed from one other; nor did they appear to resemble the other varieties of *G. leucocarpa* outside of mainland China. We collected these unusual populations from Wuliang Mountain of Jingdong county, Fenshuiling Divide of Luchun in Yunnan Province, and Mang Mountain of Yizhang County in Hunan Province, respectively.

In this study, we focus on the delimitation of the taxa of the *G. leucocarpa* group from mainland China. With phylogenetics and principal component analysis (PCA) analyses on both morphological and population genetic data, we investigate the taxonomic relationships among *G. leucocarpa* var. *yunnanensis*, *G. leucocarpa* var. *crenulata*, *G. leucocarpa* var. *pingbienensis*, and the three unusual populations mentioned above, together with samples of two forms, that is, *G. leucocarpa* var. *leucocarpa* f. *leucocarpa* and *G. leucocarpa* var. *leucocarpa* f. var. *cumingiana* (Vidal) Sleumer, both of which were recognized at the varietal level in the classification of Middleton (1991, 1993) from the Philippines and Malaysia. We address the following questions: (1) Are the three varieties and the three unusual populations within *G. leucocarpa* from mainland China distinct from one another genetically? (2) Can morphological characters be identified that unequivocally distinguish the three unusual populations from the other varieties? (3) Do the unusual populations merit taxonomic recognition? and (4) At what rank should the mainland entities currently considered all as *G. leucocarpa* be recognized?

## 2 MATERIALS AND METHODS

### 2.1 Taxon sampling

Morphological, genetic, biological, and ecological characteristics may not always agree. A broader definition of a species should incorporate several criteria, especially in considering that different lines of evidence can be available for separating a given species from others (Dantas‐Torres, 2018; Liu, 2016). In this context, we selected 11 populations from a larger pool of 84 populations of *G. leucocarpa* that were sampled across mainland China in a cpDNA phylogenetic study by Li et al. (2020). We integrated both molecular and morphological data to support the recognition of the number of species and their delimitation, with habitat information also considered. We sampled the three unusual populations (JD, MS, YLC), one population of *G. leucocarpa* var. *crenulata* (WD), one population of *G. leucocarpa* var. *pingbienensis* (DWS) collected in the vicinity of the type locality, and six populations of *G. leucocarpa* var. *yunnanensis* (10 individuals for each population were sampled, Table S2). DWS, JD, YLC, and WD were collected from Yunnan and MS was sampled from Hunan. The six representative populations of *G. leucocarpa* var. *yunnanensis* were from Yunnan (PB, DL, and YGN), Guangxi (JWS), Guangdong (DHY), and Southwest Sichuan (SGL).

To assess the monophyly of the *G. leucocarpa* group and reconstruct the phylogenetic relationships among its mainland members, we conducted phylogenetic analysis on all 11 populations above plus three adjacent‐region populations within the *G. leucocarpa* group and included an additional 62 species samples of *Gaultheria* from Lu et al. (2019). The three adjacent‐region populations included one of *G. leucocarpa* var. *yunnanensis* (HXQ from Taiwan of China), one of *G. leucocarpa* var. *leucocarpa* f. *cumingiana* (F from Davao del Sur of the Philippines), and one of *G. leucocarpa* var. *leucocarpa* f. *leucocarpa* (LYW from Pahang of Peninsular Malaysia). Two individuals of each population were sampled (Table S2). The taxonomy of *G. leucocarpa* outside of China follows the treatment of Sleumer (1967).

### 2.2 DNA extraction, gene amplification and sequencing

Four DNA genic regions (nrDNA ITS and three cpDNA regions *rpl16*, *matK*, and *trnL‐trnF*) were used for phylogenetic reconstruction of *Gaultheria* as in Fritsch et al. (2011). In addition, the cpDNA regions *rpl33‐psaJ* and *rpl32‐trnL* (Li et al., 2020), and the low‐copy nrDNA regions *AAT* (aspartate aminotransferase, Gong & Gong, 2016) and *LOC* (an intergenic region newly screened) were employed for a population genetic analysis. Total DNA from leaf samples was extracted with the cetyl trimethyl ammonium bromide (CTAB) method (Doyle & Doyle, 1987). The PCR reaction mixture contained 10 μL of PCR Master Mix (2×) (Thermo Scientific), 9 μL of nuclease‐free water, 0.5 μL of each pair of primers (10 ng/μL) (Table S3), and 1 μL of template DNA. The methods of PCR amplifications and procedures were performed as in Li et al. (2020); for primer annealing temperatures, see Table S3. The amplified products were directly sequenced with the Sanger method by using amplification primers and BigDye on an ABI 3730 capillary sequencer (Applied Biosystems).

### 2.3 Phylogenetic analysis and population genetic clusters

We downloaded 260 DNA sequences of ITS, *rpl16*, *matK* and *trnL‐trnF* from 64 species (two species of *Eubotrys* Nutt. as outgroup) based on Lu et al. (2019) from GenBank (Table S4). We generated 112 new DNA sequences from 28 samples among 14 populations of the *G. leucocarpa* group for a 92‐terminal phylogenetic analysis (Appendix S1, Table S5). Sequences were aligned with the web‐based version of MAFFT (https://mafft.cbrc.jp/alignment/server/) and manually adjusted. We reconstructed the phylogeny with maximum likelihood in RAxML 7.0.4 (Stamatakis et al., 2008), simultaneously generating 1000 bootstrap replicates under the GTRGAMMA model. To evaluate taxon/entity boundaries within *G. leucocarpa*, we compared the genetic divergence among populations of *G. leucocarpa* with the interspecific divergence among the sampled *Gaultheria* species using K2P‐distance and p‐distance, both of which were calculated by MEGA 6.0 with the default parameters and 10,000 bootstrap replicates.

The genetic structure of 110 individuals from 11 populations was analyzed with the cpDNA combined data from *rpl33‐psaJ* and *rpl32‐trnL* (Appendix S2) and the nrDNA combined data of *AAT* and *LOC* (Appendix S3, Table S6), both with a Bayesian clustering method implemented in STRUCTURE ver. 2.3.4 (Hubisz, 2010). This program assigns individuals to *K* subpopulations (clusters) based on an admixture model and a correlated allele frequencies model. To infer the “best” value of *K*, we first ran the analysis at each value of *K* from 1 to 10 with 10 replicate runs per value of *K* with a burn‐in of 10,000 and 100,000 sampled MCMC generations. The results of preliminary runs were processed with Structure Harvester (Earl & Vonholdt, 2012). These runs suggested delta*K* peaks for each of the nuclear and plastid datasets at *K* = 2. Results based on the nuclear data discriminated one genetic cluster in the MS population and another in all the remaining sampled populations. We then performed a STRUCTURE analysis on the nuclear dataset with the MS population excluded to obtain high resolution of the clusters within the remaining sampled populations, with deltaK peaks at *K* = 2 and 4 both suggested.

### 2.4 Principal component analysis with morphological and genetic data

To clarify the taxonomy of the 11 populations of *G. leucocarpa* group from mainland China, we examined our collections from the field and other herbarium specimens. Morphological characters based on habit, vegetative organs, flowers, and fruit were measured and compared. From each population, data were taken on 10 individuals randomly selected in the field and three other herbarium specimens. From these characters, 19 quantitative and six qualitative characters were used for principal component analysis. Most characters were selected on the basis of our 18‐year taxonomic investigation and other work, which found these characters to be diagnostic for species delimitation of *Gaultheria* (Fritsch & Lu, 2020; Lu et al., 2010; Middleton, 1991). The 19 quantitative characters for PCA are (1) plant height, (2) maximal width of the stem base, (3) maximal blade length of the leaf borne at the basal position of a branch, (4) maximal blade length of leaf borne at the central position of a branch, (5) maximal blade length of leaf borne at the upper position of a branch, (6) mean value of leaf blade length on a branch [formula: [(3) + (4) + (5)]/(3)], (7) maximal blade width of leaf borne at the basal position of a branch, (8) maximal blade width of leaf borne at the central position of a branch, (9) maximal blade width of leaf borne at the upper position of a branch, (10) mean value of leaf blade width on a branch [formula: [(7) + (8) + (9)]/(3)], (11) number of marginal teeth on the blade of a leaf borne at the basal position of a branch, (12) number of marginal teeth on the blade of a leaf borne at the central position of a branch, (13) number of marginal teeth on the blade of a leaf borne at the upper position of a branch, (14) mean value of the number of leaf marginal teeth of a branch [formula: [(11) + (12) + (13)]/(3)], (15) number of flowers in an inflorescence, (16) length of bract (at the pedicel base), (17) maximal length of corolla, (18) maximal width of corolla, and (19) ratio of style length to the polar axis of young fruit (Appendix S4). The six qualitative characters are (1) leaf blade texture (chartaceous or coriaceous vs. thickly coriaceous), (2) presence vs. absence of indumentum on inflorescence, (3) presence vs. absence of indumentum of bract apex margin, (4) presence vs. absence of indumentum on calyx lobe margin, (5) calyx color (green vs. green flushed with red), and (6) corolla color (light whitish green vs. light whitish green flushed with red; Appendix S4). PCA analyses were performed with two datasets, that is, morphological data, and morphological data combined with genetic mutation sites. Genetic mutation sites were generated from the data of the STRUCTURE analysis that concatenated the two chloroplast regions *rpl33‐psaJ* and *rpl32‐trnL* and the two low‐copy nrDNA regions *AAT* and *LOC* (Appendix S4) with the FactoMineR package in R 4.0.2.

## 3 RESULTS

### 3.1 Sequence alignment, phylogenetic analysis, and genetic divergence

For the four‐genic dataset of *Gaultheria*, the aligned DNA sequence matrix consisted of 4593 bp (with 35.5% GC content), of which 14.93% were variable sites and 8.99% were parsimony‐informative characters (PICs). The most variable genic region was ITS, with 24.26% variable sites and 15.53% PICs, whereas the least variable region was *rpl16*, with 12.46% variable sites and 7.18% PICs. For the datasets of the 11 populations of *G. leucocarpa* from mainland China, the aligned DNA sequence matrix of the two‐cpDNA combined dataset (*rpl33‐psaJ* and *rpl32‐trnL*) consisted of 1268 bp (with 30.7% GC content), of which 1.18% were variable sites and 1.02% were PICs, and that of two low‐copy nrDNA dataset (*AAT* and *LOC*) consisted of 1407 bp (with 48.1% GC content), of which 8.52% were variable sites and 6.89% were PICs. In addition, a 31‐bp insertion was found in the *trnL‐rpl32* region of the YLC samples, and a 65‐bp insertion was found in the *AAT* region of the MS samples.

The best phylogenetic tree of *Gaultheria* from ML recovered a topology in which the samples of *G. leucocarpa* form a monophyletic group (BP = 100%, Figure S1, Appendix S5). This group is sister to the Diplycosia clade (see Kron et al., 2020). Phylogenetic relationships among most populations were resolved with bootstrap support greater than 80%. The *G. leucocarpa* group was divided into a clade comprising samples of the MS population, *G. leucocarpa* var. *yunnanensis* (SGL), *G. leucocarpa* var. *crenulata* (WD), and *G. leucocarpa* var. *leucocarpa* f. *leucocarpa* (LYW; BP = 71%); and a clade comprising the rest of the samples (BP = 94%). Within the latter, JD, YLC, *G. leucocarpa* var. *pingbienensis* (DWS), and all populations of *G. leucocarpa* var. *yunnanensis* except SGL form one clade with strong support (BP = 87%), and another clade comprises the population of *G. leucocarpa* var. *yunnanensis* from Taiwan (HXQ) and *G. leucocarpa* var. *leucocarpa* f. *cumingiana* (F), with 100% support. The monophyly of *G. leucocarpa* var. *pingbienensis* (DWS, BP = 88%), JD (BP = 79%), MS (BP = 100%), YLC (BP = 100%), as well as that of *G. leucocarpa* var. *leucocarpa* f. *cumingiana* (F, BP = 100%), and *G. leucocarpa* var. *leucocarpa* f. *leucocarpa* (BP = 100%) were recovered. The phylogenetic relationships among the five populations of *G. leucocarpa* var. *yunnanensis*, that is, DHY, DL, JWS, PB, and YGN, were poorly resolved, but they all form a clade with high support (BP = 93%).

The K2P‐ and *P*‐distances were compared at the intraspecific level within *G. leucocarpa* and interspecific level within *Gaultheria* and found to be generally the same, and some were greater than or equal to those found between species of some *Gaultheria* (Appendix S6). The intraspecific divergences within *G. leucocarpa* of both K2P‐ and *P*‐distances ranged from 0 (stdv. 0) to 0.006 (stdv. 0.001), with an average of 0.003 (stdv. 0). The K2P and *P* interspecific distances within *Gaultheria* ranged from 0 (stdv. 0) to 0.34 (stdv. 0.003); 11% were <0.006. The K2P and *P* average interspecific divergences of four out of 13 clades (i.e., Amblyandra, Pernettya, Monoanthemona, and Myrtilloideae; see clade information in Figure S1) within *Gaultheria* were ≤0.003.

### 3.2 Population genetic structure

**FIGURE 1:**
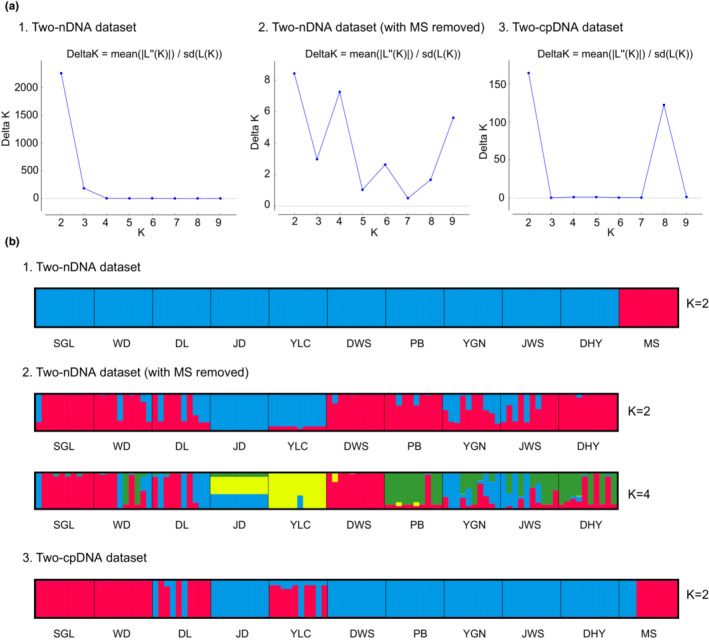
STRUCTURE analyses on the low‐copy nuclear and plastid gene datasets of the *Gaultheria leucocarpa* group from mainland China. (a‐1) The best‐fit model (Δ*K*) for the dataset based on concatenated two nuclear genes; (a‐2) the best‐fit model (Δ*K*) for the dataset based on concatenated two nuclear genes with the data of MS population removed; (a‐3) the best‐fit model (Δ*K*) for the dataset based on concatenated two‐plastid‐gene regions. (b‐1) The plot of genetic clusters of the two‐nuclear‐gene dataset of all populations at *K* = 2 (each column/grid represents a population); (b‐2) plots of genetic clusters based on the two‐nuclear‐gene dataset of all populations with MS removed at *K* = 2 and 4; (b‐3) plot of genetic clusters based on the two‐plastid‐gene dataset of all populations at *K* = 2.

**FIGURE 2:**
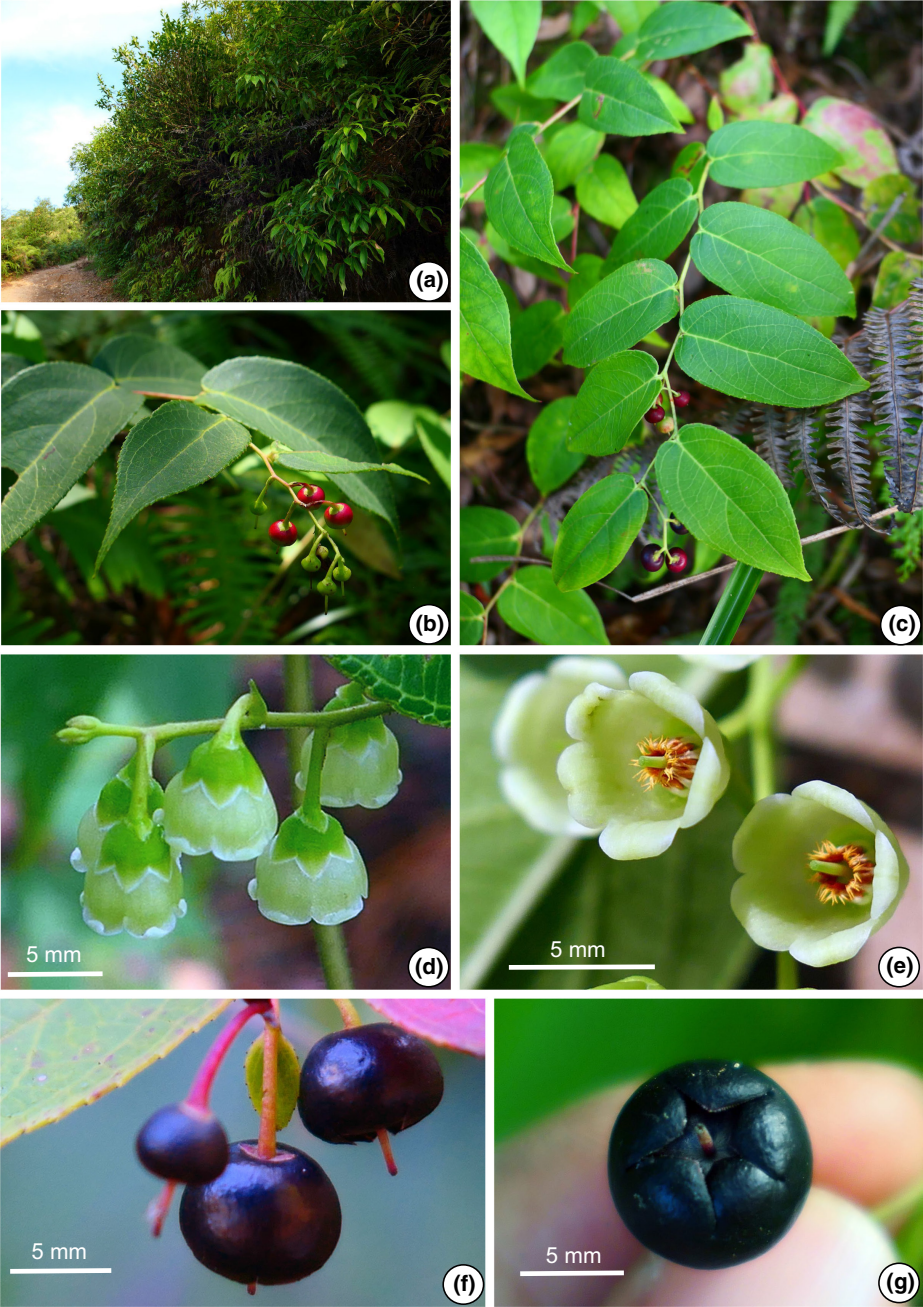
*Gaultheria crenulata* (*L. Lu* et al. *LL‐2019‐52*). (a) Habit. (b, c) Branchlets, showing leaf arrangement and oblate young red and (immature) green fruits. (d) Flowers in lateral view, showing campanulate whitish green corolla, and corolla margin rolled outward. (e) Flowers in oblique‐apical view, showing campanulate whitish green corolla. (f) Fruits in lateral view, showing oblate mature deeply bluish black mature fleshy calyces with rose red or orange fruiting pedicels. (g) Fruit in apical view, showing slightly open mature fleshy calyx and pubescent capsule. [Photos, by Y.R. Li and L. Lu].

**FIGURE 3:**
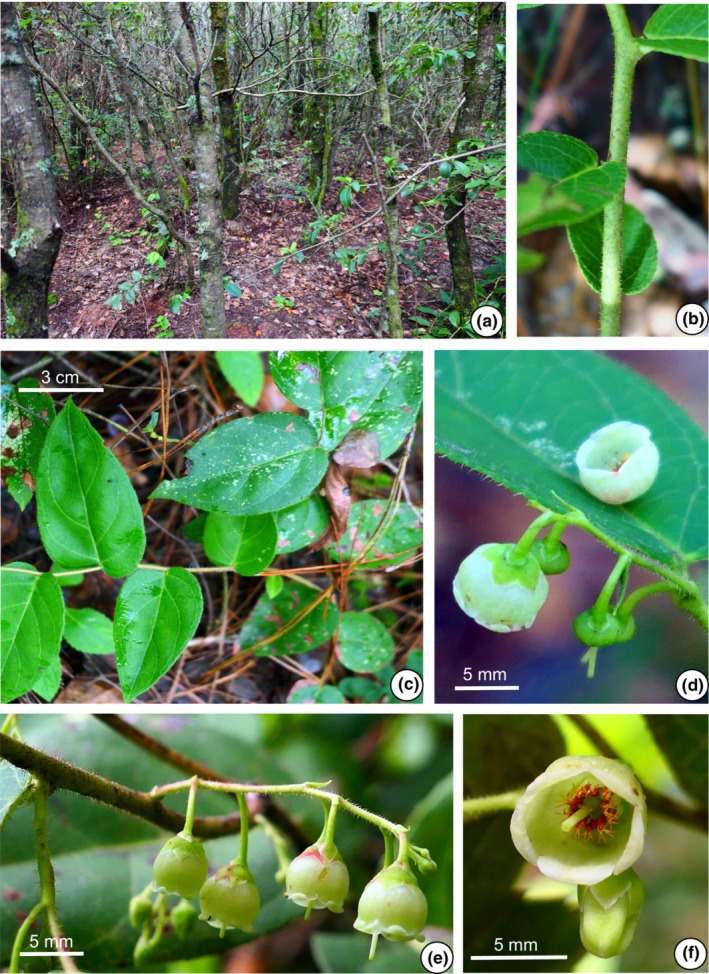
*Gaultheria crenulata* (*L. Lu* et al. *LL‐2020‐50*, population WD). (a) Habitat. (b) Branchlets, showing trichomes on stem. (c) Branchlet, showing leaf arrangement and trichomes on young branchlets. (d) Leaf and inflorescence, showing trichomes on the leaf margin, campanulate whitish green corolla, green calyces, bracts and bracteoles, and young green mature fleshy calyx. (e) Inflorescence and flowers in lateral view, showing trichomes on the rachis, and green calyces, bracts, and bracteoles occasionally flushed with rose red. (f) Flower in apical view. [Photos, by Y.R. Li and L. Lu].

Bayesian clustering analysis with STRUCTURE yielded a best‐fit model where the highest Δ*K* of the two‐gene nrDNA dataset is *K* = 2 (Δ*K* = 2255.95, Figure 1a‐1) and that of the plastid dataset is *K* = 2 (Δ*K* = 164.01, Figure 1a‐3). Two clusters were identified from the nrDNA dataset, one comprising population MS and the other comprising the remaining 10 populations (Figure 1b‐1). When the data from MS were excluded, Δ*K* was highest at *K* = 2 (Δ*K* = 8.42), and second‐highest at *K* = 4 (Δ*K* = 7.25; Figure 1a‐2). When *K* = 2, two clusters were found, one comprising *G. leucocarpa* var. *yunnanensis* (DHY, DL, JWS, PB, SGL, and YGN), *G. leucocarpa* var. *crenulata* (WD), and *G. leucocarpa* var. *pingbienensis* (DWS) and the other comprising JD and YLC. When *K* = 4, four clusters were found: one cluster included *G. leucocarpa* var. *yunnanensis* (DHY, DL, JWS, PB, SGL, and YGN) and *G. leucocarpa* var. *crenulata* (WD, with mixture of red, blue, yellow, and green as depicted in Figure 1); one cluster of *G. leucocarpa* var. *pingbienensis* (DWS, with a mixture of red and yellow); one cluster of JD (with a mixture of green, yellow, and blue); and another cluster of YLC (with a mixture of yellow and blue; Figure 1b‐2). From the plastid data, two genetic clusters were recovered: one comprises mainly *G. leucocarpa* var. *yunnanensis* populations DHY, JWS, JD, PB, YGN, and *G. leucocarpa* var. *pingbienensis* (population DWS; depicted in blue), and another comprises mainly *G. leucocarpa* var. *yunnanensis* (DL, SGL), *G. leucocarpa* var. *crenulata* (WD), MS, and YLC (depicted in red; Figure 1a‐3).

### 3.3 Comparison of morphological characters

**FIGURE 4:**
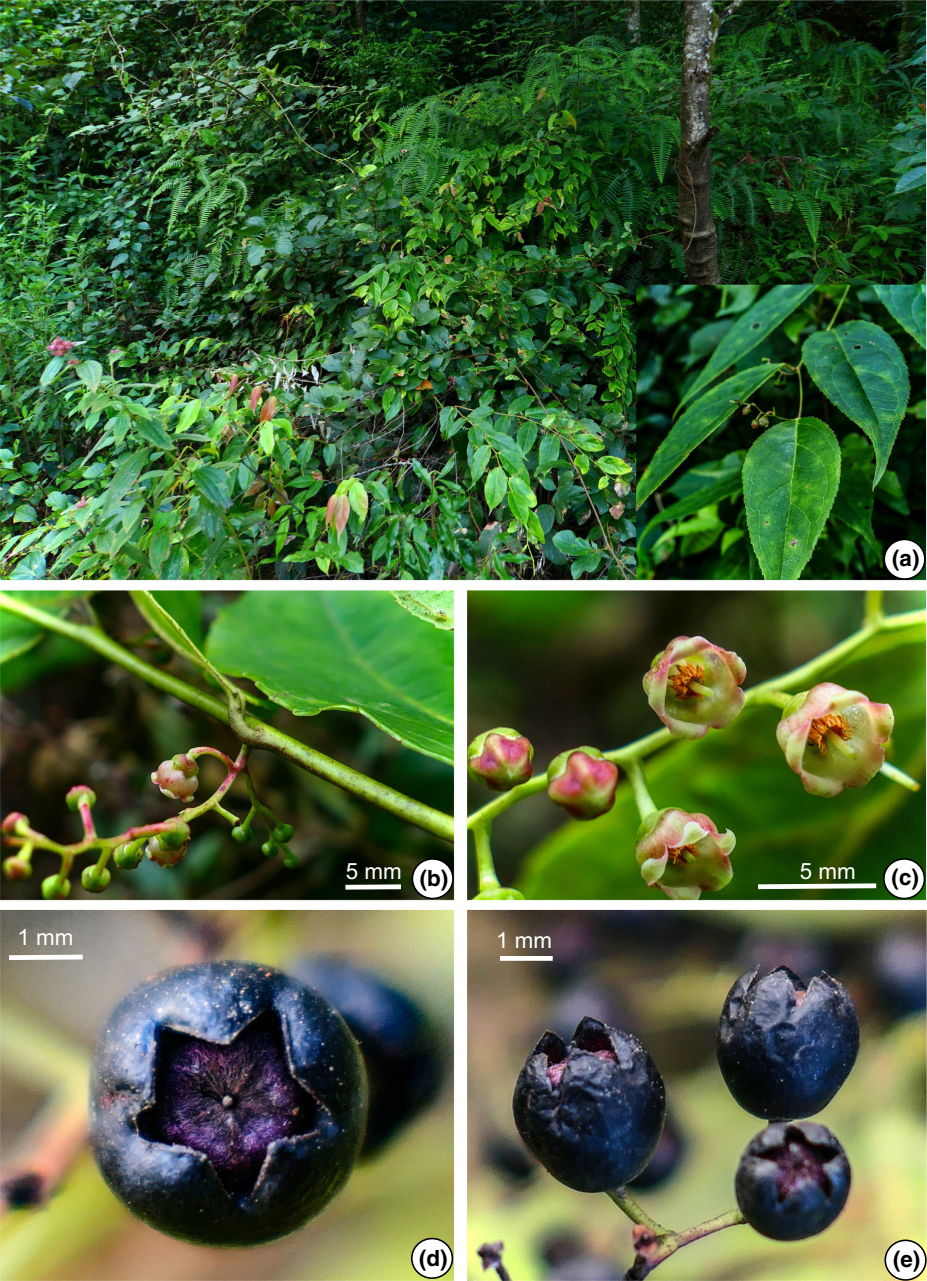
*Gaultheria luchunensis* Yi R. Li, Lu Lu & P.W. Fritsch (*L. Lu* et al. *LL‐2020‐44*). (a) Habit, and branchlets, showing leaf arrangement. (b) Inflorescence, showing bracts, bracteoles and flower pedicels. (c) Buds and flowers in apical view, showing campanulate whitish green corolla flushed with rose red. (d) Fruit in apical view, showing deeply bluish black open mature fleshy calyces and purple pubescent capsules. (e) Fruits in lateral view, showing elongate mature fleshy calyces and erect calyx lobes. [Photos, by Y.R. Li and L. Lu].

**FIGURE 5:**
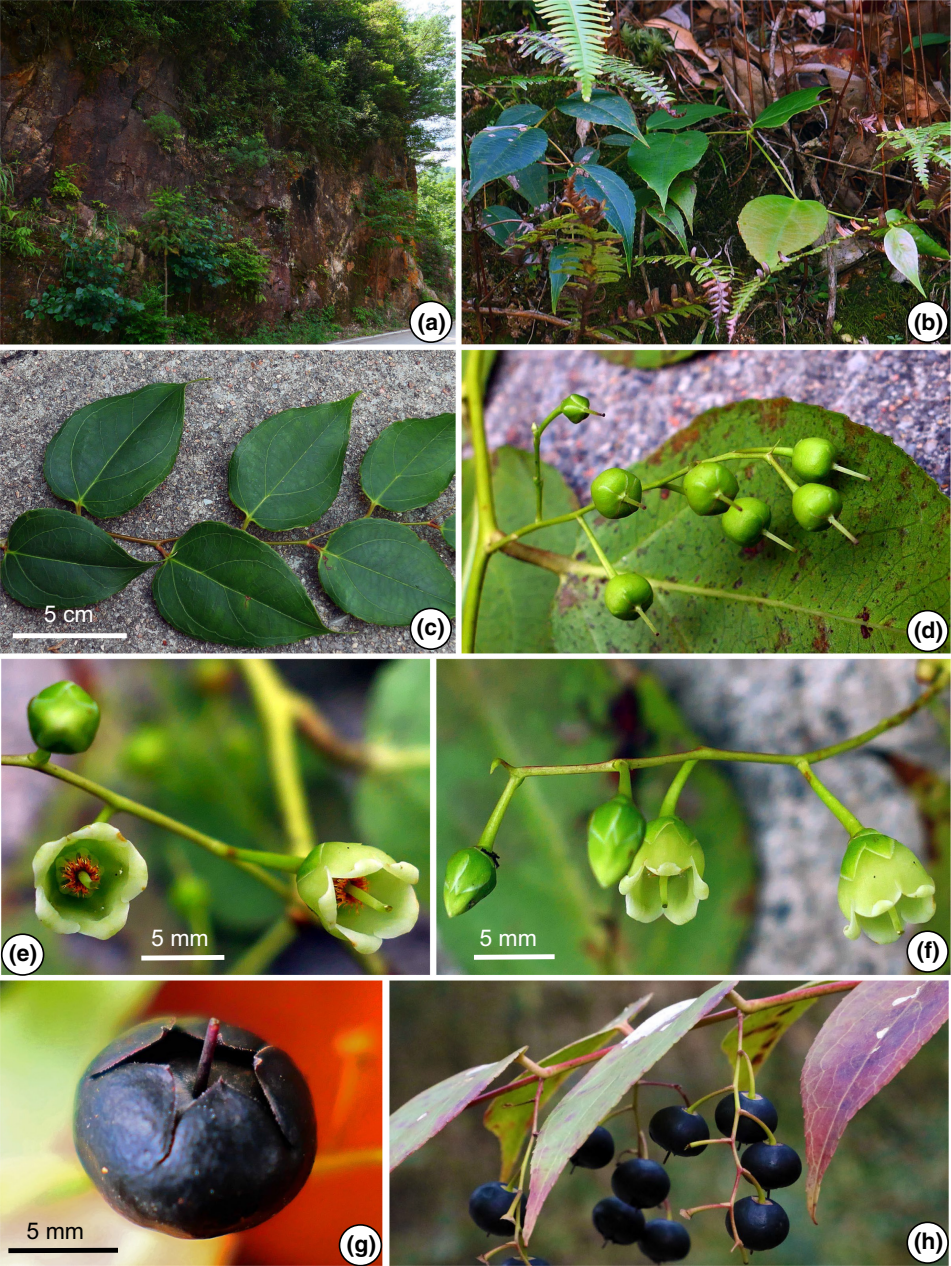
*Gaultheria mangshanensis* Yi R. Li, Lu Lu & P.W. Fritsch (*L. Lu* et al. *LL‐2019‐36*). (a) Habitat. (b–d) Branchlets. (b) Branchlets and leaf arrangement; (c) leaves, showing deep green color with light green veins. (d) Infructescence, showing spherical or elongate‐spherical green immature calyces. (e) Flowers and buds in apical view, showing narrowly campanulate whitish green corolla and long styles. (f) Flowers and buds in lateral view showing green bracts, bracteoles, and calyces. (g) Fruit in apical view showing deeply bluish black slightly open mature fleshy calyces and purple pubescent capsules. (h) Fruits in lateral view, showing subglobose or slightly oblate spheroidal mature fleshy calyces. [Photos, by Y.R. Li and L. Lu].

**FIGURE 6:**
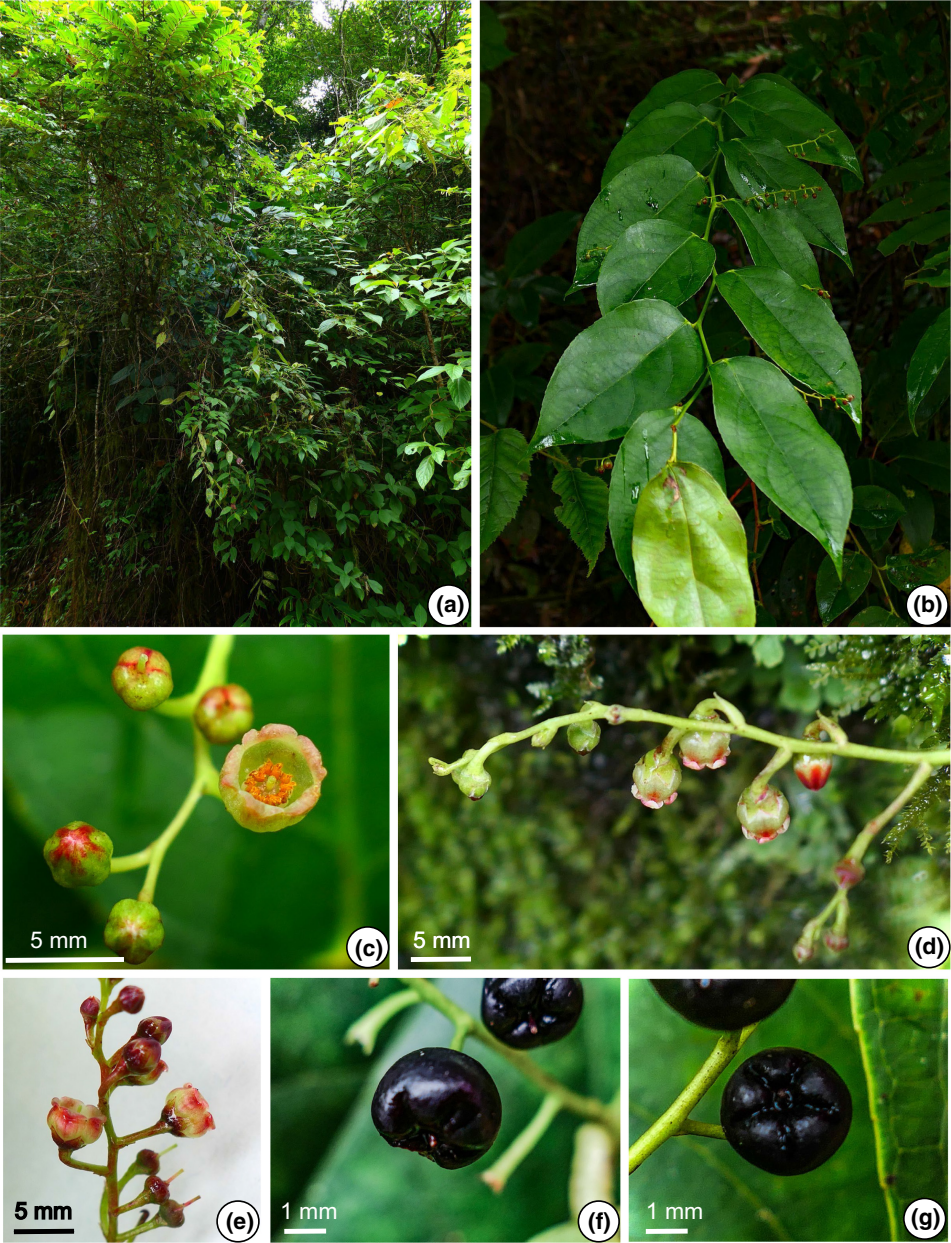
*Gaultheria pingbienensis* (C.Y. Wu ex T.Z. Xu) Yi R. Li, Lu Lu & P.W. Fritsch (*L. Lu* et al. *LL‐2020‐39*). (a) Habitat. (b) Branchlets, showing leaf arrangement and inflorescence with attached buds, flowers and immature fruits. (c) Flower buds in rose red in apical view; showing campanulate whitish green corolla with rose red margin rolled outward; immature green calyces with rose red calyx lobes. (d) Inflorescence, showing bracts and bracteoles. (e) Inflorescence in lateral view, showing rose red buds, flowers, and immature fruits with long styles. (f) Fruits in lateral view, showing deeply bluish black oblate mature fleshy calyces. (g) Fruit in apical view, showing closed mature fleshy calyces. [Photos, by Y.R. Li, and L. Lu].

**FIGURE 7:**
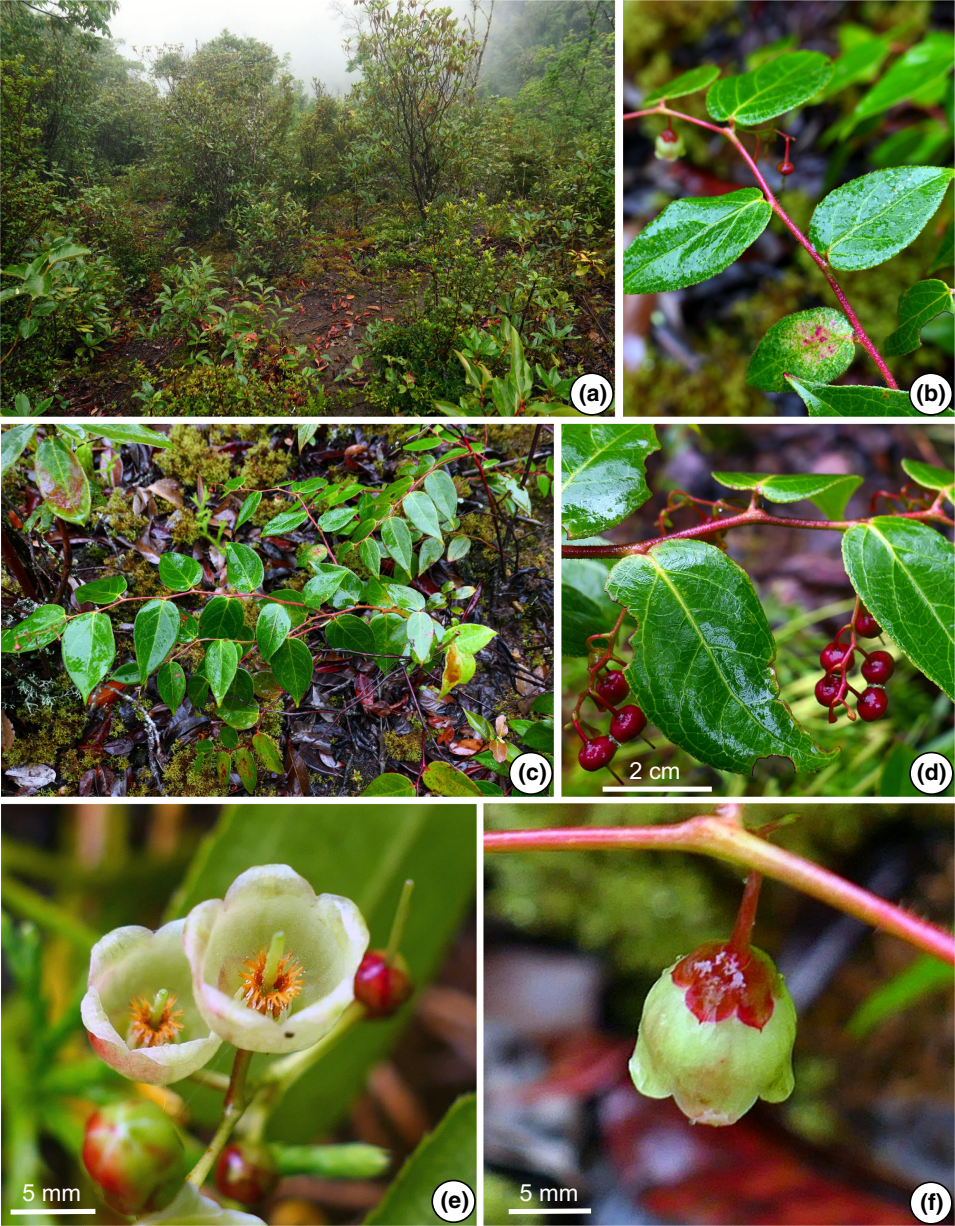
*Gaultheria wuliangshanensis* Yi R. Li, Lu Lu & P.W. Fritsch (*L. Lu* et al. *LL‐2020‐25*). (a) Habitat. (b–d) Fruiting and flowering branchlets. (b) Leaf arrangement and red stem; (c) young branchlets; (d) infructescence, showing oblate immature mature fruiting calyces. (e) Flowers and buds, showing campanulate whitish green corolla mixed with maroon, and long styles. (f) Flower in lateral view, showing maroon bracteoles and calyces. [Photos, by Y.R. Li, and L. Lu].

The morphological characters of the 11 populations of the *G. leucocarpa* group from mainland China were compared. The photographs depict six populations belonging to the *G. leucocarpa* group, which comprised the three atypical populations (JD, MS, YLC), one population of *G. leucocarpa* var. *crenulata* (WD), one population of *G. leucocarpa* var. *pingbienensis* (DWS), and one population of *G. leucocarpa* var. *yunnanensis* (DHY; Figures 2, 3, 4, 5, 6, 7). *Gaultheria leucocarpa* var. *pingbienensis* (DWS) is morphologically distinct from *G. leucocarpa* var. *yunnanensis* by height (typically 1.8–3.7 vs. 0.2–2.7 m), maximal width of the base of the stem: 7.6–15.3 mm (vs. 1.6–14.4 mm), leaf blade 8.1–11.4 × 2.7–5.4 cm (vs. 3.0–9.4 × 1.6–4.3 cm), flowers 2.7–4.2 × 2.8–4.7 mm (vs. 4.1–7.8 × 2.5–8.0 mm), corolla light whitish green flushed with red (vs. white or light whitish green), and style of young fruit 1.2–1.8 mm long (vs. 2.9–4.8 mm). YLC is similar to *G. leucocarpa* var. *pingbienensis* (DWS) in plant height and leaf texture but differs by leaf marginal teeth 0.37–0.94 mm (vs. 0.20–0.66 mm), flower buds ribbed (vs. unribbed), and fruiting calyx elongate and open (vs. oblate and closed). JD resembles populations of *G. leucocarpa* var. *yunnanensis* in leaf shape, midvein, inflorescence position, plant size, and fruit indumentum but differs by the presence of glandular‐setose trichomes (vs. absence of such trichomes), leaves thick‐coriaceous (vs. coriaceous or chartaceous), leaf marginal teeth 45–80 per side (vs. 25–45), flowers 7.2–9.2 × 7–9.3 mm (vs. 4.1–7.8 × 2.5–8.0 mm), style on young fruit 5.5–6.1 mm long (vs. 2.9–4.8 mm long), and margins of bracts, bracteoles, and calyx lobe apices glabrous or rarely ciliolate (vs. ciliolate). MS resembles populations of *G. leucocarpa* var. *yunnanensis* in its flexuous stem, inflorescence position, calyx shape and color, and fruit indumentum but differs by leaf blade base deeply cordate and apex acute (vs. base shallowly cordate‐ovate and apex acuminate), secondary veins 2 or 3 pairs (vs. 3 or 4), leaf texture thickly coriaceous (vs. coriaceous or chartaceous), and length of style on young fruit 4.2–5.6 mm (vs. 2.9–4.8 mm). Populations of *G. leucocarpa* var. *crenulata* (WD) resemble those of *G. leucocarpa* var. *yunnanensis* in plant size, leaf size and shape, and calyx shape and color, but differ by the presence of glandular‐setulose trichomes on branches, leaves, and inflorescences (vs. absence), and leaf marginal teeth 56–73 per side (vs. 25–45).

### 3.4 Principal component analysis of morphological and genetic data

**FIGURE 8:**
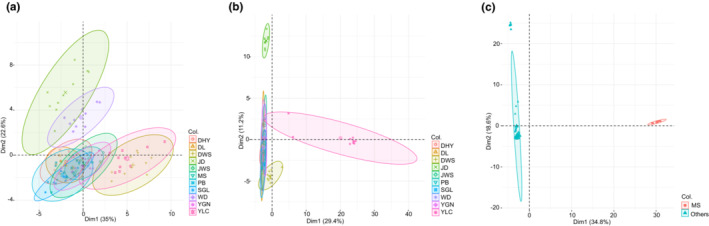
Genetic and morphological data used for the principal component analysis of the *Gaultheria leucocarpa* group from mainland China. (a) Principal component analysis (PCA) plot of all populations based on morphological data. (b) The PCA plots of all populations based on concatenated morphological and genetic data with the MS population removed. (c) The PCA plots of MS and all other populations (i.e., treating MS as one group and all other populations as another) based on concatenated morphological and genetic data. Ellipses represent 95% confidence intervals, which show 95% high‐density regions for normal distributions representing each morphological group (or morphological plus genetic data group).

Parameters with the highest coefficient values were selected on the basis of the strongest correlation with each component in the PCA analyses (Figure 8a–c; 95% confidence intervals depicted with ellipses). The first two principal components derived from morphological characters explain 57.6% of the variation. PC1 (35% relative contribution) was determined mainly by characters of plant height, maximal width of the stem base, leaf size (width and length of blade), and corolla color, whereas PC2 (22.6%) was determined mainly by the number of leaf marginal teeth, corolla size, and ratio of style length to the polar axis of young fruit (Figure 8a). The ordination shows that *G. leucocarpa* var. *pingbienensis* (DWS), JD, and YLC tend to segregate from *G. leucocarpa* var. *crenulata* (WD). When we excluded the data of MS, we found higher resolution among the other populations (40.6% of the observed variables in the first two principal components; PC1 = 29.4%, PC2 = 11.2%; Figure 8b). PC1 is highly influenced by the variation in genetic data of JD and YLC, whereas PC2 is mainly influenced by the morphometric parameters with the highest coefficient values (i.e., corolla size, leaf blade texture, number of leaf marginal teeth, ratio of style length to the polar axis of young fruit, and bracts, bracteoles and calyces with or without marginal cilia). When we treated MS as one group and the other populations as another, 53.4% of the observed variables were found in the first two principal components (contribution: PC1 = 34.8%, PC2 = 18.6%; Figure 8c). Thus, MS segregates well along PC1.

## 4 DISCUSSION

The populations of the *G. leucocarpa* group from mainland China were found not only to be morphologically variable but also to form morphological and genetic clusters, suggesting that a taxonomic revision is warranted. The morphological differences were found in both vegetative and reproductive characters, in particular in the morphology of the bracts, bracteoles, and calyces (Figure S2). Moreover, the infraspecific K2P‐ and *P*‐distances were found to be equal to or higher than interspecific distances of several clades within *Gaultheria*, suggesting that species‐level recognition, rather than the current varietal recognition, is justified. *G. leucocarpa* var. *pingbienensis* (DWS), as well as MS, JD, and YLC, each formed its own genetic ancestry palette. Resolution in the combination of morphological and genetic data is greater than that of morphological data only, and the ranking of PC1 and PC2 can be distinctly divided into four categories, together further separating the populations YLC, MS, JD, and *G. leucocarpa* var. *pingbienensis* (DWS) but failing to distinguish WD (*G. leucocarpa* var. *crenulata*) from the populations of *G. leucocarpa* var. *yunnanensis*. In addition, YLC, MS, JD, and *G. leucocarpa* var. *pingbienensis* (DWS) mainly occur on high ridges or damp slopes under undisturbed forest with high endemicity, vs. *G. leucocarpa* var. *yunnanensis*, which occurs on open slopes in full or partial sun and mostly in disturbed secondary forest. Based on the sum of our four‐year field surveys and the results of analyses presented here with both morphological and genetic data, we consider JD, MS, YLC, *G. leucocarpa* var. *pingbienensis* (DWS), and *G. leucocarpa* var. *yunnanensis* each recognizable as separate taxa, all at the rank of species.

Although neither *G. leucocarpa* var. *crenulata* nor the SGL population of *G. leucocarpa* var. *yunnanensis* formed a clade with the other five populations of *G. leucocarpa* var. *yunnanensis*, there is still insufficient evidence for their taxonomic recognition apart from *G. leucocarpa* var. *yunnanensis*. In addition, the topology appears to be strongly biased toward the cpDNA result rather than that of nrDNA because of the poor resolution in ITS (see Lu et al., 2010). In the STRUCTURE analysis at the population level, although cpDNA grouped *G. leucocarpa* var. *crenulata* in a cluster with MS and YLC, nrDNA grouped it with all populations from *G. leucocarpa* var. *yunnanensis* in the same cluster. The conflict between cpDNA and nrDNA could result from reticulate evolution, considered to be likely occurring among species of Chinese *Gaultheria* (Lu et al., 2010, 2019). *G. leucocarpa* var. *crenulata* (and SGL) is therefore suspected to be involved in cpDNA introgression. Furthermore, these populations are not distinct from *G. leucocarpa* var. *yunnanensis* when morphological and genetic data are combined in PCA.

*Gaultheria leucocarpa* var. *crenulata* differs from *G. leucocarpa* var. *yunnanensis* only by the presence (vs. absence) of dense glandular‐setose trichomes on stems, branchlets, leaves, and inflorescences, which was used as diagnostic characters to distinguish these taxa in Fang and Stevens (2005). Based on field investigation from 2017 to 2021, we found that the indumentum characters on stems, branches, and leaves are sporadic and found in the mature stage if growing in shaded and moist habitats, or in the seedling or young stage in many populations of *G. leucocarpa* var. *yunnanensis*. This is consistent with the description of *G. leucocarpa* in Mustaqim and Setiawan (2020) in stating that hairs are usually present in juvenile plants. Trichomes commonly act as a barrier to protect plants from herbivores, ultraviolet radiation, or excessive transpiration (Johnson, 1975; Mauricio & Rausher, 1997), or as secretory or accumulative organs to attract or repel insects and other organisms (Dalin et al., 2008). Instead of genetics, the presence of indumentum might be influenced more by environmental factors and therefore be a poor character for taxonomic delimitation of *G. leucocarpa*. Alternatively, the trichome character might be a simple genetic polymorphism within a species. In any case, as a result we elevate *G. leucocarpa* var. *yunnanensis* to the species level with resurrection of *G. crenulata* as the species name based on the principle of priority (*G. crenulata* Kurz; barcode Isotype K 000228003!). We henceforth refer to this taxon as *G. crenulata*.

We collected *G. leucocarpa* var. *pingbienensis* (DWS, Figure 6) in the vicinity of the holotype; this entity is clearly distinct from *G. crenulata* on the basis of both morphological and genetic data. *G. leucocarpa* var. *pingbienensis* is a historically problematic variety. Fang and Stevens (2005) treated it as a synonym of *G. leucocarpa* var. *yunnanensis*, and Fritsch et al. (2008) tentatively as an accepted name with the synonym *G. leucocarpa* var. *yunnanensis*. Nevertheless, *G. leucocarpa* var. *pingbienensis* has the largest plants, largest leaves, and smallest flowers within *G. leucocarpa*, with corollas that are light whitish green flushed with red (vs. white or light whitish green in *G. crenulata*). Based on these characters and its genetic distinctness, we elevate it to the rank of species (*G. pingbienensis*). The protologue of Hsu (1981) was incomplete because only leaf morphology was described. The sheets of the type (K.M. Feng 4827; barcode holotype KUN 0482955! and Isotype KUN 0778843!) were found to be ambiguous, without flowers and with leaf features intermediate between those of *G. crenulata* and our material that we consider to be *G. pingbienensis* (the leaves are as small as those of *G. crenulata*, but the leaf texture and veins are similar to *G. pingbienensis*). Perhaps leaf texture and veins were poor characters to distinguish these taxa, and additional collections need to be made to understand the nature of morphological variation and the taxonomic status of this species more completely. Here, we designate another specimen as epitype for *G. pingbienensis* which contains representative leaves, flowers, and young and mature fruits.

The population YLC was also found to be both morphologically and genetically distinct. It resembles *G. pingbienensis* in the field because of similarity in habitat (shaded and moist broadleaf forest), habit, plant height, and leaf texture. However, they are distinguishable by differences in the size of leaf marginal teeth, shape of flower buds, and fruit shape and openness. Shape and openness of the calyx at fruiting have been commonly neglected in the taxonomy of *Gaultheria* species because of deformation of the fruit after pressing and drying on herbarium specimens but were found to be key characters in the taxonomy of the core East Asian clade, especially that of *G*. series *Trichophyllae* (Fritsch et al., 2011, 2015; Fritsch & Lu, 2020; Lu et al., 2010). We found that the fruiting calyces of *G. pingbienensis* are oblate spheroidal and closed, whereas those of the YLC population are elongate‐spheroidal and open. Moreover, the length of the leaf marginal teeth of YLC is 0.2–0.66 mm vs. that of *G. pingbienensis* at 0.37–0.94 mm. We therefore recognize YLC as the newly described species *Gaultheria luchunensis*.

Another population from Yunnan, JD, is here recognized as the newly described species *Gaultheria wuliangshanensis* (Figure 7). Although the monophyly of *G. wuliangshanensis* is not strongly supported (BP = 77%, Figure S1), both the population genetic structure and PCA analyses based on the concatenated morphological‐genetic data support its separation from all other studied populations. Unlike *G. crenulata*, this species tends to grow on high ridges in undisturbed forest and has thickly coriaceous leaves and a substantially longer fruiting style. Moreover, *G. wuliangshanensis* has several features unique to the mainland *G. leucocarpa* group, such as the largest corollas (7.2–9.2 × 7–9.3 mm), the most broadly ovate calyx lobes, and glabrous or very sparsely ciliolate margins of the bracts, bracteoles, and calyx lobes. Marginal indumentum of the calyx lobes, an overlooked character in Middleton's classification of *Gaultheria* (Middleton, 1991), was later found to be useful as a character for species identification in *G*. series *Trichophyllae* (Fritsch et al., 2015, 2016; Fritsch & Lu, 2020), and now it is clear that it is also useful for the *G. leucocarpa* group. Variation in the indumentum of the calyx and corolla was also found to be good characters for diagnosing *G. leucocarpa* var. *hirta* (Mustaqim & Setiawan, 2020; Sleumer, 1967).

Finally, we here recognize the population MS as the newly described species *Gaultheria mangshanensis*, endemic to Hunan. In our phylogenetic analysis, the monophyly of *G. mangshanensis* is strongly supported and sister to *G. leucocarpa* var. *leucocarpa* f. *leucocarpa*. The population genetic structure and PCA analyses both place *G. mangshanensis* well away from all other studied populations. The morphology of this species is similar to *G. crenulata* in plant height and leaf marginal teeth, but differs in leaf texture and shape, that is, the leaf blades are more thickly coriaceous and deeply cordate at the base and caudate‐acuminate at the apex (vs. leaf blade base ovate and apex attenuate at the apex), and secondary veins only 2 or 3 pairs (vs. 3 or 4). In addition, the persistent style is longer (4.2–5.6 mm vs. 2.9–4.8 mm). *G. mangshanensis* tends to grow on high ridges in undisturbed forest, unlike *G. crenulata*, which grows on open slopes in secondary forest.

Based on morphology, DNA sequences, and multiple field surveys from 2017 to 2021, we describe three new species (*G. luchunensis*, *G. mangshanensis*, and *G. wuliangshanensis*), make one new combination (*G. pingbienensis*) with a new epitype, and resurrect *G. crenulata*. Marginal cilia of the calyx lobes, bracts, and bracteoles, and fruit shape and openness until now have been overlooked in herbarium observations but uncovered as substantially diagnostic characters for the varieties within *G. leucocarpa* distributed in mainland China. This is the first paper to study in detail the taxonomy of the *G. leucocarpa* group from mainland China. This work has resulted in an increase of species within the Gymnobotrys clade of *Gaultheria*. Based on phylogenetics, we found *G. leucocarpa* var. *yunnanensis* from Taiwan to be sister to *G. leucocarpa* var. *leucocarpa* f. *cumingiana*. Our genetic evidence supports the conclusion of Alafag and Napaldet (2022) that elevated *G. leucocarpa* var. *cumingiana* to the species level as *G. cumingiana*. Our preliminary examination of the *G. leucocarpa* group from Taiwan and southeastern Asia suggests that higher species diversity exists than current taxonomic treatments indicate. The *G. leucocarpa* group therefore merits further taxonomic studies with increase in both genomes and international sampling, both of which may ultimately aid in understanding the number, rank, and circumscription of taxa in the group.

## 5 TAXONOMIC TREATMENT

**Key to the species of the *G. leucocarpa* clade in mainland China**

1. Margins of calyces, bracts, and bracteoles eciliolate; corolla 7.2–9.2 × 7.0–9.3 mm; style of young fruit 5.5–6.1 mm long…………………………………….. ***G. wuliangshanensis***

1. Margins of calyces, bracts, and bracteoles ciliolate; corolla 2.7–7.8 × 2.5–8.0 mm; style of young fruit 1.2–5.6 mm long.

2. Leaf blades mostly 8.1–12.9 × 2.8–5.4 cm; corolla light whitish green flushed with red, 2.7–4.5 × 2.8–5.0 mm; style of young fruit 1.2–1.9 mm long.
  3. Fruiting calyces closed and covering the capsule…………… ***G. pingbienensis***
  3. Fruiting calyces open and not covering the capsule………….. ***G. luchunensis***
2. Leaf blades mostly 3.0–9.4 × 2.8–4.5 cm; corolla light whitish green, 4.1–7.8 × 2.5–8.0 mm; style of young fruit 2.9–5.6 mm long.
  4. Leaf blade base obtuse to rounded, apex acuminate, leaf texture chartaceous or coriaceous, secondary veins 3 or 4 pairs; style of young fruit 2.9–4.8 mm long……………………………………. ***G. crenulata***
  4. Leaf blade base cordate, apex caudate‐acuminate, leaf texture thickly coriaceous, secondary veins 2 or 3 pairs; style of young fruit 4.2–5.6 mm long…………………………………………………………… ***G. mangshanensis***

### 5.1 Gaultheria crenulata

Kurz, J. Bot. 11:195. 1873. ≡ *Gaultheria leucocarpa* Blume var. *crenulata* (Kurz) T.Z. Xu, Acta Bot. Yunnan. 3:429. 1981. Type: CHINA. Yunnan: Tengchong, Hotha, 15 August 1868, *D.J. Anderson s.n*. (holotype: CAL, not seen, Dr. K. Biswas mentioned that he was unable to find the type of *Gaultheria crenulata* Kurz in the Calcutta herbarium in 1941, see Merrill, 1941). Figures 2 and 3, Figures S2A (1–3) and S2B (1–3).

= *Gaultheria leucocarpa* var. *hirsuta* (D. Fang & N.K. Liang) T.Z. Xu, Acta Bot. Yunnan. 3:428. 1981. Basionym: *Gaultheria yunnanensis* (Franch.) Rehder var. *hirsuta* D. Fang & N.K. Liang, Acta Phytotax. Sin. 15(2):112. 1977. Type: CHINA. Guangxi: Guiping Xian, Zijin, Yuanan, *N.K. Liang & D. Fang 10,748* (holotype: GXMI‐050387 [photo!]; isotypes: GXMI‐050389 [photo!]).

*= Gaultheria leucocarpa* var. *yunnanensis* (Franch.) T.Z. Xu & R.C. Fang, Novon 9:166. 1999. Basionym: *Vaccinium yunnanense* Franch., J. Bot. (Morot) 9:368. 1895. ≡ *Gaultheria yunnanensis* (Franch.) Rehder, J. Arnold Arbor. 15:282. 1934. Type: CHINA. Yunnan: Tchen‐fong‐chan [Cheng‐feng‐shan], *P.J.M. Delavay 3069* (holotype: L‐0007113 [photo!], P‐00715775 [photo!]; isotype: P‐00715777 [photo!]).

**Chinese Name:** 滇白珠 dian bai zhu.

**Description: Shrubs**, prostrate or ascending, 0.2–2.5 (−2.7) m tall. Current‐year branchlets pale green or red, glabrous or setose‐glandular‐hirsute. Internodes averaging 1.7–3.2 cm long. **Leaves:** with wintergreen odor when crushed; alternate, petioles 2.5–5.2 mm long, abaxially glabrous or occasionally with sparse setae, adaxially glabrous, margin entire; blades ovate to ovate‐lanceolate, 3.0–9.4 × 1.6–4.5 cm, 2.0–2.2 times as long as wide, coriaceous or chartaceous, abaxially dull whitish green, glabrous or setose‐glandular‐hirsute and white‐puberulent, adaxially glossy deep green, glabrous, setose‐glandular‐hirsute and white‐puberulent, or rarely with sparse brownish glandular trichomes on midvein proximally, midvein abaxially raised, secondary veins 3 or 4 pairs, arising along midvein, with proximal veins becoming faint or anastomosing before reaching apex, adaxially slightly depressed, base obtuse to rounded, margin serrulate throughout, apex acuminate, marginal teeth (setae) 25–45 (73) per side. **Inflorescences**: axillary racemes 1.5–15.0 cm long, 2–14‐flowered; rachis flexuous, slender, red or green when fresh, glabrous or rarely glandular‐setulose‐hirsute and white‐puberulent; bracts narrowly triangular to triangular, 1.8–3.1 mm long, persistent, glabrous, margin white‐ciliolate, apex acute. Pedicel 4.2–6.1 mm long, glabrous; bracteoles 2, opposite or subopposite, borne at apex of pedicel near calyx, lanceolate‐triangular, 1.2–2.0 mm long, base cordate, apex acute‐acuminate, margin white‐ciliolate. **Flowers:** calyx green, lobes 5, deltoid‐ovate, 1.2–1.6 × 1.6–2.1 mm, adaxially glabrous, apex acute, margin white‐ciliolate. Corolla light whitish green, campanulate, glabrous, 4.1–7.8 × 2.5–8.0 mm; lobes 5, short but distinct, deltoid, somewhat recurved, 1.6–1.8 × 1.9–3.4 mm. Stamens 10; filaments curved, spindle‐shaped, 1.3–1.8 mm long; anther body 1.6–2.3 mm long, awns 2 per theca, 0.3–0.4 mm long. Style pale green, 3.4–4.5 mm long. Stigma green. Fruiting pedicel 9.2–11.6 mm long, glabrous. **Fruits:** calyx slightly open, fleshy, oblate spheroidal, subglobose, or slightly prolate spheroidal, 5.8–7.8 × 6.3–8.1 mm. Young fruits green, starting to turn red and turning dark purple to black and becoming fleshy when mature, style persistent, young fruiting style 2.9–4.8 mm long. Capsule dark purple or blackish purple, densely white‐puberulent, covered by calyx lobes. **Seeds**: light brown or light tawny brown, triangular‐obovoid, ca. 0.5 mm in diam.

**Phenology:** Flowering July–September; fruiting September–December.

**Habitat and distribution:** On slopes and along roadsides in full or partial sun in disturbed secondary forest, pine forests with regenerating understory, or occasionally on ridges; growing with *Camellia* L., *Cunninghamia* R. Br, *Dicranopteris* Bernh., *Lyonia* Nutt., *Pinus* L., *Quercus* L., and *Rhododendron* L.; sedimentary rocks; red and yellow sandy soil; 200–3300 m elevation. Distributed in the provinces of Fujian, Guangdong, Guangxi, Guizhou, Hubei, Hunan, Jiangxi, Chongqing, Sichuan, Taiwan, and Yunnan, China.

### 5.2 Gaultheria luchunensis

Yi R. Li, Lu Lu & P.W. Fritsch sp. nov. Type: CHINA. Yunnan, Honghe Prefecture, Luchun County, Daxing Town, Watershed to Geka Section, the Divide of Yuanyang County and Luchun County, 22°59′19″ N, 102°27′29″ E, 1890–2026 m, 1 Sep 2020, *L. Lu*, *Y.R. Li*, *Y.L. Xu*, *X.J. Cheng*, *H.H. Shen*, *LL‐2020‐44* (holotype: KUN‐1523867! and isotype: KUN‐1523866!). Figure 4, Figure S2C (1–3).

**Chinese Name:** 绿春白珠 lü chun bai zhu.

**Diagnosis:** *G. luchunensis* is similar to *G. pingbienensis* in plant height and leaf texture but differs by length of leaf marginal teeth (0.37–0.94 mm vs. 0.20–0.66 mm) and in particular fruit shape and openness. The mature fruiting calyxes are oblate spheroidal or subglobose and closed, and covering the capsules in *G. pingbienensis*, whereas they are subglobose or prolate spheroidal and open, and not covering the capsules in *G. luchunensis*.

**Description:** Shrubs or small trees, prostrate or ascending, 0.8–3.5 m tall. Current‐year branchlets pale green or red, glabrous, rarely densely brownish‐setose‐glandular‐hirsute. Internodes averaging ca. 2.9–4.3 cm long. **Leaves:** with wintergreen odor when crushed; alternate, petioles 6–9.7 mm long, abaxially glabrous, adaxially glabrous, margin entire; blades ovate to ovate‐lanceolate, 8.8–12.9 × 2.8–5.0 cm, 3.1–3.3 times as long as wide, chartaceous or rarely coriaceous, abaxially dull whitish green, rarely setose‐glandular‐hirsute, adaxially glossy deep green, glabrous, midvein abaxially raised, secondary veins 3 or 4 pairs, arising along midvein with proximal veins becoming faint or anastomosing before reaching apex, adaxially slightly depressed, base ovate to ovate‐lanceolate, margin serrulate throughout, apex caudate‐attenuate, marginal teeth (setae) 27–45 per side. **Inflorescences:** axillary racemes 3.0–7.5 cm long, 6–17‐flowered; rachis flexuous, slender, red or green when fresh, glabrous or rarely glandular‐setulose‐hirsute and white‐puberulent; bracts narrowly triangular to triangular, 1.2–3.5 mm long, persistent, glabrous, margin white‐ciliolate, apex acute. Pedicel 3.1–4.4 mm long, glabrous; bracteoles 2, opposite or subopposite, borne at apex of pedicel, lanceolate‐triangular, 0.9–1.3 mm long, base cordate, apex acute‐acuminate, margin white‐ciliolate. **Flowers:** calyx green or green flushed with red, lobes 5, deltoid‐ovate, 0.8–1.1 × 0.8–1.2 mm, adaxially glabrous, apex acute, margin white‐ciliolate. Corolla light whitish green flushed with rose red, campanulate, glabrous, 3.1–4.5 × 3.6–5.0 mm; lobes 5, short but distinct, deltoid, somewhat recurved, 0.9–1.0 × 0.9–1.2 mm. Stamens 10; filaments curved, spindle‐shaped, 0.6–0.9 mm long; anther body 0.7–1.1 mm long, awns 2 per theca, 0.1–0.2 mm long. Style pale green, 1.6–1.9 mm long. Stigma green. Fruiting pedicel 2.6–5.4 mm long, glabrous. **Fruits:** calyx open, fleshy, subglobose to prolate spheroidal, 6.7–8.5 × 5.9–7.9 mm. Young fruits green, starting to turn red and turning dark purple to black and becoming fleshy when mature, style persistent, young fruiting style 1.2–1.9 mm long. Capsule dark purple, densely white‐puberulent, not covered by calyx lobes. **Seeds**: light brown or light tawny brown, triangular‐obovoid, ca. 0.5 mm in diam.

**Etymology:** The epithet “*luchunensis*” is derived from the location of the holotype in Luchun County, Yunnan Province.

**Phenology:** Flowering June–August; fruiting August–December.

**Habitat and distribution:** On road banks and in broadleaf undisturbed forests; growing with *Castanopsis* (D. Don) Spach, *Ilex* L., *Lasianthus* Jack, *Rubus* L., *Schima* Reinw. ex Blume, *Smilax* L., and *Viburnum* L. in forest humus or red and yellow sandy soil; 1100–2026 m elevation. Distributed in Yunnan Province, China.

### 5.3 Gaultheria mangshanensis

Yi R. Li, Lu Lu & P.W. Fritsch sp. nov. Type: CHINA. Hunan, Chenzhou City, Yizhang County, Mang Mountain National Forest Park, 24°57′09″ N, 112°57′59″ E, 1220–1442 m, 15 Aug 2019, *L. Lu*, *Y.R. Li*, *G.H. Li*, *Y.Q. Chen*, *LL‐2019‐36* (holotype: KUN‐1523860! and isotype: KUN‐1523861!). Figure 5, Figure S2D (1–3).

**Chinese Name:** 莽山白珠 mang shan bai zhu.

**Diagnosis:** *Gaultheria mangshanensis* is similar to *G. crenulata* in plant height, leaf margin, and mature branches but different in habits that *G. mangshanensis* tends to occur on high ridges of hills under undisturbed forest. In this species, the leaf texture is thickly coriaceous, the persistent style is generally long, the leaf blade base is deeply cordate, the leaf blade apex is caudate‐acuminate, and secondary veins are only with 2 or 3 pairs.

**Description:** Shrubs, prostrate or ascending, 0.2–1.5 m tall. Current‐year branchlets pale green or red, glabrous, rarely densely brownish‐setose‐glandular‐hirsute. Internodes averaging 2.9–3.8 cm long. **Leaves:** with wintergreen odor when crushed; alternate, petioles 5.6–8.5 mm long, abaxially glabrous, adaxially glabrous, margin entire; blades ovate to ovate‐lanceolate, 5.6–8.9 × 2.8–4.5 cm, 1.9–2 times as long as wide, thickly coriaceous, abaxially dull whitish green, rarely brownish glandular‐hirsute, adaxially glossy deep green, glabrous, midvein abaxially raised, secondary veins 2 or 3 pairs, arising along midvein with proximal veins becoming faint or anastomosing before reaching apex, adaxially slightly depressed, base cordate, margin serrulate throughout, apex caudate‐acuminate, marginal teeth (setae) 16–36 per side. **Inflorescences:** axillary racemes 2.8–5.4 cm long, 4–8‐flowered; rachis flexuous, slender, red or green when fresh, glabrous or rarely glandular‐setulose‐hirsute and white‐puberulent; bracts, narrowly triangular to triangular, 1.2–2.9 mm long, persistent, glabrous, margin white‐ciliolate, apex acute. Pedicel 4.6–6.1 mm long, glabrous; bracteoles 2, opposite or subopposite, borne at apex pedicel, lanceolate‐triangular, 1.3–2.0 mm long, base cordate, apex acute‐acuminate, margin white‐ciliolate. **Flowers:** calyx green or green flushed with white, lobes 5, deltoid‐ovate, 1.6–2.5 × 1.9–2.9 mm, adaxially glabrous, apex acute, margin white‐ciliolate. Corolla light whitish green, campanulate, glabrous, 5.8–7.5 × 5.5–6.8 mm; lobes 5, short but distinct, deltoid, somewhat recurved, 0.8–1.2 × 1.1–1.2 mm. Stamens 10; filaments curved, spindle‐shaped, 2.3–2.5 mm long; anther body 1.8–2.3 mm long, awns 2 per theca, 0.5–0.9 mm long. Style pale green, ca. 2.1 mm long. Stigma brown or brownish green. Fruiting pedicel 4.2–5.1 mm long, glabrous. **Fruits:** calyx slightly open, fleshy, subglobose or slightly oblate spheroidal, 7.8–9.8 × 8.5–11.7 mm; Young fruits green, starting to turn red and turning dark purple to black and becoming fleshy when mature, style persistent, young fruiting style 4.2–5.6 mm long. Capsule dark purple, densely white‐puberulent, covered by calyx lobes. **Seeds:** light brown or light tawny brown, triangular‐obovoid, ca. 5 mm in diam.

**Phenology:** Flowering May–July; fruiting August–December.

**Etymology:** “*mangshanensis*” is derived from the location of the holotype in Mang Mountain, Hunan Province.

**Habitat and distribution:** On road banks, on ridges in undisturbed forests; growing with *Castanopsis*, *Celastrus* L., *Cunninghamia*, *Cyclobalanopsis* Oerst., *Dicranopteris*, *Exbucklandia* R.W.Br. and *Pinus*; forest humus or yellow sandy soil; 1220–1442 m elevation. Distributed in Hunan Province, China.

### 5.4 Gaultheria pingbienensis

C.Y. Wu ex T.Z. Xu. Yi R. Li, Lu Lu & P.W. Fritsch comb. & stat. nov. Basionym: *Gaultheria leucocarpa* var. *pingbienensis* C.Y. Wu ex T.Z. Xu, Acta Bot. Yunnan. 3:429. 1981. Type: CHINA. Pingbian, Laojian peak of Ada Kou in Shiban town, ca. 2180 m, 10 Oct 1954, *Feng G.M. 4827*. holotype: KUN‐1208603! and epitype, here designated: Yunnan, Dawei Mountain of Pingbian County, 22°55′39″ N, 103°41′25″ E, 1895–2074 m, 31 Aug 2020, *L. Lu*, *Y.R. Li*, *Y.L. Xu*, *X.J. Cheng*, *H.H. Shen*, *LL‐2020‐39* (KUN‐1523864! and KUN‐1523865!). Figure 6, Figure S2E (1–3).

**Chinese Name:** 屏边白珠 ping bian bai zhu.

**Diagnosis:** *Gaultheria pingbienensis* is similar to *G. crenulata* in its pubescent ovary, fruit shape, and secondary veins but differs by larger height (1.8–3.7 m tall vs. 0.2–2.7 m), larger leaf blade (8.1–11.4 × 2.7–5.4 cm vs. 3.0–9.4 × 1.6–4.5 cm), and smaller flowers (2.7–4.2 × 2.8–4.7 mm vs. 4.1–7.8 × 2.5–8.0 mm) with light whitish green flushed with red color rather than white or light whitish green (of *G. crenulata*).

**Description:** Shrubs or small trees, prostrate or ascending, 1.8–3.7 m tall. Current‐year branchlets pale green or red, glabrous, rarely densely brownish‐setose‐glandular‐hirsute. Internodes averaging 3.1–3.9 cm long. **Leaves:** with wintergreen odor when crushed; alternate, petioles 5.8–9.5 mm long, abaxially glabrous, adaxially glabrous, margin entire; blades ovate to ovate‐lanceolate, 8.1–11.4 × 2.7–5.4 cm, 2.1–4 times as long as wide, chartaceous, rarely thick, abaxially dull whitish green, rarely setose‐glandular‐hirsute, adaxially glossy deep green, glabrous, midvein abaxially raised, secondary veins 3 or 4 pairs, arising along midvein with proximal veins becoming faint or anastomosing before reaching the apex, adaxially slightly depressed, base cordate to auriculate‐cordate, margin serrulate throughout, toward the apex of leaf, apex acuminate or caudate‐attenuate, marginal teeth (setae) 20–39 per side. **Inflorescences:** axillary racemes 3.5–8.5 cm long, 4–13‐flowered; rachis flexuous, slender, red or green when fresh, glabrous or rarely glandular‐setulose‐hirsute and white‐puberulent; bracts narrowly triangular to triangular, 1.9–5.2 mm long, persistent, glabrous, margin white‐ciliolate, apex acute. Pedicel 4.4–7.5 mm long, glabrous; bracteoles 2, beneath the calyx, opposite or subopposite, attached to distal pedicel near calyx, lanceolate‐triangular, 1.1–1.6 mm long, base cordate, apex acute‐acuminate, margin white‐ciliolate. **Flowers:** calyx green or green flushed with red, lobes 5, deltoid‐ovate, 1.0–1.3 × 1.0–1.6 mm, adaxially glabrous, apex acute, margin white‐ciliolate. Corolla light white‐green flushed with rose red, campanulate, glabrous, 2.7–4.2 × 2.8–4.7 mm; lobes 5, short but distinct, deltoid, somewhat recurved, 0.6–1.1 × 1.1–1.3 mm. Stamens 10, filaments curved, spindle‐shaped, 0.6–0.7 mm long; anther body 0.7–1.0 mm long; awns 2 per theca, 0.1–0.2 mm long. Style pale green, 1.2–1.7 mm long. Stigma green. Fruiting pedicel 4.3–6.7 mm long, glabrous. **Fruits:** calyx closed, fleshy, oblate spheroidal to subglobose, 6.1–7.2 × 7.3–8.3 mm. Young fruits green, starting to turn red by September and turning dark purple to black and becoming fleshy when mature by November, style persistent, young fruit style 1.2–1.8 mm long. Capsule dark purple, densely white‐puberulent, covered by calyx lobes. Seeds: light brown or light tawny brown, triangular‐obovoid, ca. 0.5 mm in diam.

**Phenology:** Flowering June–August; fruiting August–December.

**Habitat and distribution:** On the slopes of roadsides and under broadleaved forest with undisturbed forests and in ravine; growing with *Acer* L., *Actinidia* Lindl., *Ampelopsis glandulosa* (Wall.) Momiy., *Castanopsis*, *Dichroa* Lour., *Euonymus* L., *Machilus* Rumph. ex Nees, and *Vaccinium* L.; forest humus soil; red and yellow sandy soil; 1400–2074 m elevation. Distributed in Yunnan Province, China.

### 5.5 Gaultheria wuliangshanensis

Yi R. Li, Lu Lu & P.W. Fritsch sp. nov. Type: CHINA. Yunnan, Pu'er City, Jingdong County, Wuliang Mountain Nature Reserve, Huangcaoling Village, 24°21′48″ N, 100°44′47″ E, 2300 m, 17 Aug 2020, *L. Lu*, *Y.R. Li*, *Y.L. Xu*, *X.J. Cheng*, *H.H. Shen*, *LL‐2020‐25* (holotype: KUN‐1523862! and isotype: KUN‐1523863!). Figure 7, Figure S2F (1–3).

**Chinese Name:** 无量山白珠 wu liang shan bai zhu.

**Diagnosis:** *Gaultheria wuliangshanensis* is similar to *G. crenulata* in plant height and leaf size; they both also have ovate to ovate‐lanceolate leaves blades with auriculate‐cordate base, and secondary veins with 3 or 4 pairs. However, *G. wuliangshanensis* differs in its thickly coriaceous leaf blade texture and the persistent style is longer. Moreover, *G. wuliangshanensis* has dense brownish glandular trichomes on branchlets and its leaf margins are sparsely hispid and serrulate. Notably, it has the largest corolla among all taxa of *G. leucocarpa* from mainland China (7.2–9.2 × 7–9.3 mm). The broadly ovate calyx lobes and the glabrous or rarely ciliolate margins of bracts, bracteoles, and calyces are also unique.

**Description:** Shrubs, prostrate or ascending, 0.4–2.8 m tall. Current‐year branchlets pale green or red, glabrous or densely brownish‐setose‐glandular‐hirsute. Internodes averaging 2.0–3.1 cm long. **Leaves:** with wintergreen odor when crushed; alternate, petioles 2.8–5.4 mm long, abaxially glabrous or occasionally with sparse setae, adaxially glabrous, margin entire; blades ovate to ovate‐lanceolate, 5.0–9.7 × 2.4–4.2 cm, 2.1–2.3 times as long as wide, thickly coriaceous, abaxially dull whitish green, sparsely brownish glandular‐hirsute, adaxially glossy deep green, glabrous, midvein abaxially raised, secondary veins 3 or 4 pairs, arising along midvein with proximal veins becoming faint or anastomosing before reaching the apex, adaxially slightly depressed, base auriculate‐cordate, margin serrulate throughout, toward the apex of leaf, apex acuminate or caudate‐attenuate, marginal teeth (setae) 45–80 per side, each ending in a very short thick glandular point. **Inflorescences:** axillary racemes 2.9–5.7 cm long, 3–10‐flowered; rachis flexuous, slender, red or green when fresh, glabrous or rarely glandular‐setulose‐hirsute and white‐puberulent; bracts narrowly triangular to triangular, 1.4–4.9 mm long, persistent, glabrous, margin glabrous, apex acute. Pedicel 3.5–6.3 mm long, glabrous; bracteoles 2, beneath the calyx, opposite or subopposite, attached to distal pedicel near calyx, lanceolate‐triangular, 0.8–1.3 mm long, base cordate, apex acute‐acuminate, margin glabrous. **Flowers:** calyx green and sometimes maroon, lobes 5, broadly ovate, 1.2–1.3 × 1.9–2.2 mm, adaxially glabrous, apex acute, margin glabrous. Corolla light whitish green (sometimes flushed with maroon), campanulate, glabrous, 7.2–9.2 × 7–9.3 mm; lobes 5, short but distinct, broadly ovate, somewhat recurved, 2.1–2.6 × 1.8–2.2 mm. Stamens 10, filaments curved, spindle‐shaped, 2.3–2.9 mm long; anther body 2.1–2.2 mm long, awns 2 per theca. 1.2–1.4 mm long. Style pale green, 5.1–5.5 mm long. Stigma green. Fruiting pedicel 5.2–6.6 mm long, glabrous. Fruits: calyx slightly open, fleshy, subglobose or slightly oblate spheroidal, margin glabrous. Young fruits green, subglobose or slightly oblate spheroidal, mature not seen, starting to turn red by September, style persistent, young fruit style 5.5–6.1 mm long. Capsule with densely white puberulence, covered by calyx lobes. **Seeds:** light brown or light tawny brown, triangular‐obovoid, ca. 0.5 mm in diam.

**Etymology:** The epithet “*wuliangshanensis*” is derived from the location of the holotype on Wuliang Mountain, Jingdong County, Yunnan Province.

**Phenology:** Flowering May–August; fruiting August–November.

**Habitat and distribution:** On slopes and ridges in undisturbed forests; growing with *Lyonia*, *Pinus*, *Pteridium* Gled. ex Scop., *Rhododendron*, *Vaccinium*, and *Yushania* P.C. Keng; forest humus soil; yellow sandy soil; ca. 2300 m elevation. Distributed in Yunnan Province, China.

The additional specimens examined of the five species described above are provided in Appendix S7.

## AUTHOR CONTRIBUTIONS

**Yi‐Rong Li:** Formal analysis (lead); methodology (equal); resources (equal); writing – original draft (lead). **Yan‐Ling Xu:** Data curation (equal); formal analysis (equal); resources (equal). **Peter W. Fritsch:** Funding acquisition (equal); writing – review and editing (lead). **Lu Lu:** Formal analysis (equal); funding acquisition (lead); resources (lead); writing – original draft (equal).

## CONFLICT OF INTEREST STATEMENT

The authors declare no conflict of interest.

## Supporting information


Appendix S1

Appendix S2

Appendix S3

Appendix S4

Appendix S5

Appendix S6

Appendix S7

Figure S1

Figure S2

Tables S1–S6

## Data Availability

All datasets generated and analyzed for this study are provided within Appendices [Link], [Link], Tables S1–S6, and Figures S1 and S2. Final DNA sequence assembly uploaded to GenBank (National Center for Biotechnology Information, http://www.ncbi.nlm.nih.gov/). The datasets that associated with this study are available at DRYAD (datadryad.org) with https://doi.org/10.5061/dryad.bzkh189c8.
