# Supplementary material for: Patterns of genetic variation and morphology support the recognition of five species in the Gaultheria leucocarpa Blume (Ericaceae) group from mainland China
Source: Ecol Evol. 2023 Jun 9;13(6):e10178. doi: 10.1002/ece3.10178 (PMC10251198; doi:10.1002/ece3.10178)
Supplement: Supplementary file 7 — Appendix S7 [file ECE3-13-e10178-s008.docx]

**Additionally examined specimens of the five species in the *Gaultheria leucocarpa* Blume (Ericaceae) group from mainland China**

**1 *Gaultheria crenulata*** Kurz

**Additional specimens examined: China. Chongqin:** Jinfoshan Mountain, 29°04′33″N, 107°11′78″E, 1151 m, 30 Jul 2019, *L. Lu et al. LL-2019-06* (KUN); Fengjie, Zhuyuan Town, Longchi Village to Huaji Village, 31°14′50″N, 109°17′06″E, 788 m, 3 Aug 2019, *L. Lu et al. LL-2019-09* (KUN). **Fujian:** Longyan, Shanghang, Gutian Town, Shangfu village, 25°15′48″N, 116°51′33″E, 839 m, 19 Aug 2019, *L. Lu et al. LL-2019-42* (KUN); Longyan, Liancheng, Juxi Town, Tieshan Luodi Village, 25°27′20″N, 116°47′16″E, 918 m, 20 Aug 2019, *L. Lu et al. LL-2019-45* (KUN); Longyan, Xinluo District, Yongfu Town, Hongjian Mountain, 25°03′11″N, 117°09′42″E, 773 m, 21 Aug 2019, *L. Lu et al. LL-2019-47* (KUN); Zhangzhou, Pinghe, Jiufeng Town, Xitou Village to Fayuan Temple, 24°12′56″N, 117°06′14″E, 1091 m, 22 Aug 2019, *L. Lu et al. LL-2019-51* (KUN). **Guangdong:** Heyuan, Liyuan, Qutan Village, Zhankeng to Xizitang Road, 24°29′17″N, 114°44′06″E, 847 m, 25 Aug 2019, *L. Lu et al. LL-2019-52* (KUN); Shaoguan, Nanxiong, Baishun Town, Dongkeng Village, 25°10′24″N, 114°03′42″E, 676 m, 27 Aug 2019, *L. Lu et al. LL-2019-54* (KUN); Shaoguan, Lechang, Wushan Town, Xiaoli Village to Xiaoguan Township, 25°21′54″N, 113°30′26″E, 821 m, 27 Aug 2019, *L. Lu et al. LL-2019-55* (KUN); Shaoguan, Luyuan, Nanling National Forest Park, 24°54′33″N, 113°02′54″E, 914 m, 28 Aug 2019, *L. Lu et al. LL-2019-57* (KUN); Shaoguan, Luyuan, Luoyang Town, Tianluokeng Tianjing Mountain National Forest Park, 24°41′57″N, 113°01′16″E, 728 m, 28 Aug 2019, *L. Lu et al. LL-2019-58* (KUN); Lianzhou, Lianshan, Wo Dong Town, Carp Tail Village, 24°48′00″N, 112°03′35″E, 473 m, 29 Aug 2019, *L. Lu et al. LL-2019-60* (KUN); Yunfu, Luoding, Yungai Mountain Forest Park, 22°36′48″N, 111°11′52″E, 1055 m, 1 Sep 2019, *L. Lu et al. LL-2019-64* (KUN). **Guangxi:** Baise, Leye, Gantian, 22°55′10″N, 103°41′56″E, 1020 m, 11 Nov 2011, *J. Huang et al.* *16CS14106* (GXMG); Baise, Leye, Youping Township, Bianli Village, 24°53′26″N, 106°35′40″E, 866 m, 15 Nov 2012, *Z.Z. Lan et al. 451028121115048LY* (GXMG); Baise, Leye, Lusha Township, Jiulong Village, 24°39′55″N, 106°22′30″E, 1585 m, 15 Oct 2014, *X.S. Huang et al.* *451028141025006LY* (GXMG); Baise, Jingxi, Longbang Township, 22°53′07″N, 106°19′14″E, 844 m, 11 Dec 2012, *Y.D. Peng et al. 451025121211076LY* (GXMG); Baise, Napo, Defu Reserve, 23°16′58″N, 105°46′34″E, 1280 m, 27 Dec 2012, *D.X. Nong et al.* *451026121227030LY* (GXMG); Baise, Tianlin, Cenwanglao Mountain, 24°28′34″N, 106°23′03″E, 1800 m, 22 Jul 2013, Tianlin Expedition *451029130722010* (GXMG); Baise, Debao, Hongnipo Forest Farm, 23°28′05″N, 106°42′21″E, 883 m, 20 Oct 2015, *Debao Expedition 45102415102009LY* (IBK); Guilin, Quanzhou, Caiwan Town, around the Tianhu Reservoir, 26°4′49″N, 110°49′09″E, 616 m, 12 Aug 2013, *Quanzhou Expedition 450324130812015LY* (IBK); Guilin, Lingui, Longsheng, the junction of Longji Town, 25°40′16″N, 110°3′49″E, 720 m, 13 Aug 2014, *Longsheng Expedition 450328140813062LY* (IBK); Guilin, Ziyuan, Liangshui Township, Baishi Village, 26°00′09″N, 110°23′52″E, 736 m, 21 Jul 2015, *Ziyuan Expedition 450329150721036LY* (IBK); Guanyang, Dongjing Township, 25°18′13″N, 110°50′20″E, 662 m, 05 Aug 2015, *Guanyang Expedition 450327150805011LY* (GXMG); Guilin, Lingui, Huangsha Township, 25°36′23″N, 109°57′48″E, 814 m, 20 Aug 2015, *Lingui Expedition 450322150820002LY* (GXMG); Guilin, Ziyuan, Laoshan of Yinzhu Village, 26°15′18″N, 110°33′24″E, 1830 m, 25 Oct 2015, *Ziyuan Expedition 450329151024048LY* (IBK); Guilin, Gongcheng, Shuizhuping, Xiling Township, 25°04′49″N, 110°41′02″E, 689 m, 12 May 2015, *Gongcheng Expedition 450332160512020LY* (IBK); Guilin, Yangshuo, Xingping Town, Longjing River, 24°58′36″N, 110°36′01″E, 660 m, 9 Nov 2018, *Yangshuo Expedition 450321181109033LY* (IBK); Guilin, Lipu, Changtan river of Xinping Town, 24°24′45″N, 110°32′44″E, 1027 m, 12 Nov 2018, *Lipu Expedition 450331181112024LY* (IBK); Hechi, Luocheng, Baotan Township, Pingying Village, 25°04′38″N, 108°44′32″E, 767 m, 8 Nov 2013, *Luocheng Expedition 451225131108041LY* (GXM); Hechi, Huanjiang, Minglun Town, Mengdong Village, 25°11′00″N, 108°21′24″E, 770 m, 28 Jul 2013, *Huanjiang Expedition 451226130728030LY* (IBK); Hechi, Donglan, Lanmu Township, Nongtai Village, 24°26′48″N, 107°17′52″E, 990 m, 29 Nov 2018, *B.Y.* *Huang et al.* *451224181129010LY* (GXM); Hechi, Nandang, Lihu Township, Huaili Village, 25°07′08″N, 107°41′30″E, 746 m, 9 Aug 2017, *Nandang Expedition 451221170809015LY* (GXMG); Hechi, Nandang, Chengguan Town, 24°58′54″N, 107°30′45″E, 930 m, 3 Sep 2018, *Nandang Expedition 451221180903035LY* (GXMG); Baise, Napo, Defu Village, 23°18′58″N, 105°47′55″E, 1278 m, 20 Aug 2020, *L. Lu et al. LL-2020-27* (KUN); Hechi, Huanjiang, Jiuwanshan National Nature Reserve, 25°11′53″N, 108°34′27″E, 605 m, 22 Aug 2020, *L. Lu et al. LL-2020-28* (KUN); Liuzhou, Rongshui, Yuanbaoshan National Nature Reserve, 25°22′59″N, 109°07′33″E, 410 m, 23 Aug 2020, *L. Lu et al. LL-2020-29* (KUN); Guilin, Wantian, Hebei Village, 25°31′46″N, 109°59′53″E, 775 m, 24 Aug 2020, *L. Lu et al. LL-2020-30* (KUN); Laibing, Anxiu, Yuomeng Village, 25°05′12″N, 110°13′31″E, 841 m, 25 Aug 2020, *L. Lu et al. LL-2020-32* (KUN). **Guizhou:** Anshun, Ziyun, Bandang Town, Bandang Village, 25°53′05″N, 106°12′25″E, 1087 m, 6 Jun 2017, *X.Q. Hou 520425170606320LY* (GZT). Bijie, Dafang, Xinglong Township, Lijiagou of Guomu Village, 27°26′22″N, 105°39′02″E, 1563 m, 26 Jun 2015, *J. Song 522422150626006LY* (GZT); Bijie, Hezhang, Xingfa Township, 26°59′52″N, 104°44′39″E, [no elev.], 26 Sep 2014, *F. Liu 522428140926034LY* (GZT); Bijie, Qixingguan District, Hengtai Town, 27°25′20″N, 105°26′22″E, 1415 m, 8 Jul 2016, *S.Y. Peng 522401160708009LY* (GZT); Bijie, Weining, Yancang Town, Xiangtang Village, 26°36′55″N, 104°31′02″E, [no elev.], 25 Sep 2014, *J.Q. Wang 522427140925423LY* (GZT); Bijie, Zhijin, Maochang Town, Dazhai Village, 26°34′03″N, 106°02′13″E, 1305 m, 19 Oct 2015, *Q.S. Ji 522425151019009LY* (GZT); Guiyang, Wudang District, Yangchang Township, 26°51′46″N, 106°54′53″E, 1045 m, 27 Oct 2013, *M.C. Wang 520112131027101LY* (GYB); Liupanshui, Pugu Township, Xinxing Village, 26°03′19″N, 104°41′47″E, 1965 m, 17 Jul 2014, *Y.G. Gao & P.Z. Qu 520222140717006LY*(GZT); Qiandongnan Prefecture, Huangping, Jiuzhou Town, 26°11′59″N, 106°09′57″E, 1299 m, *23 Mar 2017, M.C. Wang 520402170323109LY* (GZT); Qiandongnan Prefecture, Rongjiang, Sanjiang Township, Sige Village, 25°56′57″N, 108°17′54″E, 428 m, 22 Apr 2019, *S.G.* *Yang et al. 522632190422603LY* (GZT); Qiandongnan Prefecture, Shibing, Shuangjing Town, 27°08′22″N, 108°11′09″E, 679 m, 18 Sep 2015, *S.G. Chen 522623150918360LY* (GZT); Qiannan Prefecture, Longli, Houhougou, Longshan Town, 26°24′20″N, 106°57′41″E, 1007 m, 13 Apr 2015, *R. Zhou 522730150413001LY* (GZT); Qiannan Prefecture, Pingtang, Zhangbu Town, 26°02′30″N, 107°04′54″E, 750 m, 15 Oct 2010, *F. Zou ZouFL0139* (KUN) ; Qianxinan Prefecture, Anlong, Xinqiao Town, Qianluotian, 25°09′35″N, 109°14′28″E, 1451 m, 28 Mar 2014, *C.Y. Deng 522328140328309LY* (GZTM); Qianxinan Prefecture, Zhenfeng, Wanlan Township, 25°22′21″N, 105°32′57″E, 1243 m, 21 Nov 2018, *Y. Jia 522325181121164LY* (GZTM); Tongren, Congjiang, Donglang Town, Kongming Village, 25°45′52″N, 108°26′15″E, 1358 m, 16 Apr 2019, *K.T. Liu 522633190416032LY* (GZTM); Tongren, Shiqian, Foding Mountain, 27°20′11″N, 108°09′18″E, 655 m, 4 Nov 2016, *J. Huang 522224161104038LY* (GZTM); Tongren, Yinjiang, Ziwei Town, 27°54′52″N, 108°35′33″E, 1013 m, 9 Aug 2019, *Y. Liu 522226190809023LY* (GZTM); Tongren, Yinjiang, Zhangyongba Village, pass of the road to Yinjiaba Village, 27°57′01″N, 108°36′26″E, 815 m, 20 Nov 2017, *L. Lu et al. LL-2017-07*(KUN); Tongren, Yinjiang, Fanjing mountains, 27°54′00″N, 108°36′19″E, 1289 m, 20 Nov 2017, *L. Lu et al. LL-2017-08*(KUN); Kaili, Shiqian, Shiwantun Village, pass of the road to Gaotang Village, 27°28′29″N, 108°11′52″E, 685 m, 21 Nov 2017, *L. Lu et al. LL-2017-9* (KUN); Kaili, Shiqian, Yaoshang Village, pass of the road to Shuanglong Village, 27°23′46″N, 108°03′04″E, 858 m, 21 Nov 2017, *L. Lu et al. LL-2017-10* (KUN); Kaili, Leishan, Donggangpo Village, Leishan national natural reserve, 26°22′10″N, 108°16′53″E, 1269 m, 22 Nov 2017, *L. Lu et al. LL-2017-12* (KUN); Duyun, Chayuan Reservoir, Doupeng mountain, 26°20′12″N, 107°29′40″E, 889 m, 22 Nov 2017, *L. Lu et al. LL-2017-13* (KUN); Pingtang, Baiyun Village, pass of the road to Baiyun Village, 25°52′31″N, 107°05′13″E, 1038 m, 23 Nov 2017, *L. Lu et al. LL-2017-14* (KUN); Pingtang, Zhangbu Village, pass of the road to Pingshan Village, 26°04′27″N, 107°05′19″E, 914 m, 23 Nov 2017, *L. Lu et al. LL-2017-15* (KUN); Duyun, Guiding, pass of the road to Yuanshan Village, 26°26′04″N, 107°09′25″E , 1004 m, 23 Nov 2017, *L. Lu et al. LL-2017-16* (KUN); Duyun, Longli, pass of the road to Longli Grassland Scenic Area, 26°24′04″N, 106°54′44″E , 1541 m, 24 Nov 2017, *L. Lu et al. LL-2017-17* (KUN); Guiyang, Huaxi. Maling Village, 26°16′35″N, 106°37′49″E, 1067 m, 24 Nov 2017, *L. Lu et al. LL-2017-18* (KUN); Duyun, Changba. 26°00′42″N, 106°21′53″E, 1392 m, 25 Nov 2017, *L. Lu et al. LL-2017-19* (KUN); Xinyi, Xinren, Chenjiagou Village, 25°26′54″N, 105°23′05″E, 1307 m, 26 Nov 2017, *L. Lu et al. LL-2017-20* (KUN). **Hubei:** Enshi, Lichuan, Maoba Town, Shiban Village, 30°03′35″N, 109°03′52″E, 822 m, 1 Aug 2019, *L. Lu et al.* *LL-2019-07* (KUN); Enshi, Lichuan, Moudao Town, Tie Feng Village, 30°26′19″N, 108°40′16″E, 1446 m, 2 Aug 2019, *L. Lu et al. LL-2019-08* (KUN); Enshi, Laifeng, Dahe Town, Lingshuixi Village to Sugimuxi Village, 29°27′47″N, 109°06′38″E, 588 m, 5 Aug 2019, *L. Lu et al. LL-2019-15* (KUN). **Hunan:** Chenzhou, Guidong, Lengshuigou of Shangzhuang Village, 25°54′06″N, 114°00′31″E, 1305 m, 28 Oct 2013, *K.M. Liu LXP03-02465* (HNNU); Xiangxi Prefecture, Huayuan, Jiwei Town, 28°20′32″N, 109°27′02″E, 833 m, 17 Aug 2018, *C.B. Ma & L. J. Shi HY20180817_1045* (JIU); Jishou, Aizhai Town, Yangmeng Village, 28°21′35″N, 109°32′14″E, 728 m, 17 Aug 2018, *S. Xiang & J.Z.* *Teng JS20180817_0926* (JIU); Shaoyang, Chengbu, Jintong Mountain, 26°07′51″N, 110°11′50″E, 1076 m, 14 May 2015, *D.G.* *Zhang et al.* *CB20150514_0289* (JIU); Shaoyang, Suining, Huangsang Nature Reserve, 26°22′01″N, 110°02′08″E, 596 m, 2 Sep 2015, *K.G. Li &* *D.G. Zhang SN20150902_0354* (JIU); Xiangxi Prefecture, Huayuan, Makechang town, 28°25′17″N, 109°29′48″E, 809 m, 7 Aug 2019, *L. Lu et al. LL-2019-17* (KUN); Huaihua, Zhijiang, Muyexi Town, Yueyitang Village, 27°32′34″N, 109°33′51″E, 796 m, 8 Aug 2019, *L. Lu et al. LL-2019-20* (KUN); Huaihua, Jingzhou, Aoshang Town, Xiangdama Village, 26°47′51″N, 109°37′56″E, 370 m, 9 Aug 2019, *L. Lu et al. LL-2019-29* (KUN); Huaihua, Tongdao, Wanfo Town, Mudjiao Village, 26°17′50″N, 109°55′21″E, 480 m, 9 Aug 2019, *L. Lu et al. LL-2019-30* (KUN); Shaoyang, Chengbu, Shankou Town, Lizhazhai Village, 26°21′14″N, 110°16′32″E, 588 m, 10 Aug 2019, *L. Lu et al. LL-2019-31* (KUN); Shaoyang, Wugang, Longxi Town, Wangjiaqiao Village, Yunshan National Forest Park, 26°41′04″N, 110°38′45″E, 504 m, 11 Aug 2019, *L. Lu et al. LL-2019-32* (KUN); Yongzhou, Shuangpai Town, Yangmingshan National Forest Park, 26°00′38″N, 111°54′56″E, 1057 m, 12 Aug 2019, *L. Lu et al. LL-2019-34* (KUN); Yongzhou, Daoxian, Shouyan Town, Kongshuyan Village, Dupangling National Nature Reserve, 25°34′10″N, 111°23′09″E, 557 m, 12 Aug 2019, *L. Lu et al. LL-2019-35* (KUN); Chenzhou, Guidong, Shatian Town, Qingshan Village, 25°52′22″N, 113°48′05″E, 798 m, 16 Aug 2019, *L. Lu et al. LL-2019-37* (KUN). **Jiangxi:** Ganzhou, Chongyi, Qiyun Mountain, 25°49′27″N, 114°01′22″E, 717 m, 22 Aug 2014, *R.P. Kuang LXP03-05666* (HNNU); Pingxiang, Luxi, Guoditan, 27°25′17″N, 113°52′41″E, 463 m, 18 Oct 2015, *D.G. Zhang LXP-06-2493* (JIU); Jian, Suichuan, Dafen Town, Miling to Linyang Village, 26°14′40″N, 114°09′16″E, 548 m, 16 Aug 2019, *L. Lu et al. LL-2019-38* (KUN); Jian, Jinggang Mountain, 26°34′17″N, 114°09′16″E, 828 m, 17 Aug 2019, *L. Lu et al. LL-2019-39* (KUN); Ganzhou, Longnan, Yangcun Town, Duntou Village, Jiulian Mountain, 26°34′17″N, 114°29′55″E, 735 m, 18 Aug 2019, *L. Lu et al. LL-2019-40* (KUN); Ganzhou, Xunwu, Jitan Town, Xiangshan Village, 24°55′22″N, 115°50′13″E, 974 m, 19 Aug 2019, *L. Lu et al. LL-2019-41* (KUN). **Sichuan:** Luzhou, Xuyong, Longfeng Township, Siping Village, 28°09′07″N, 105°17′08″E, [no elev.], 12 Oct 2013, *W.B. Ju & H.N. Deng HGX13838* (CDBI); Xichang, Lushan Mountain, 27°49′49″N, 102°15′34″E, 1924 m, 7 Nov 2017, *L. Lu et al. LL-2017-01*(KUN); Butuo, Longtan Village, Yakou Hills, 27°17′52″N, 102°49′36″E, 1810 m, 09 Nov 2017, *L. Lu et al. LL-2017-02*(KUN); Xichang, Daqing Town, 27°44′44″N, 102°20′20″E, 2045 m, 4 Aug 2020, *L. Lu et al. LL-2020-04* (KUN); Liangshan Prefecture, Ganluo, Daqing Town, Azhai Village. 28°53′51″N, 102°39′52″E, 1875 m, 5 Aug 2020, *L. Lu et al. LL-2020-05* (KUN); Liangshan Prefecture, Huidong, Qiantang Town, 26°45′17″N, 102°50′05″E, 2307 m, 6 Aug 2020, *L. Lu et al. LL-2020-07* (KUN). **Yunnan:** Yuanjiang, Yinyuan Town, Wubulu Reservoir, 23°22′47″N, 101°56′31″E, 2010 m, 7 Jun 2012, *Yuanjiang Expedition 5304280553* (IMDY); Pingbian, Xinxian Township, Benchmarking Qingxian Township, 23°07′56″N, 103°30′56″E, 1657 m, 7 Jul 2012, Pingbian Chinese *Medicine Expedition 5325230690* (IMDY); Pingbian, Yuping Town, Adakou Xiaozhai, 22°56′24″N, 103°41′19″E, 1760 m, 24 Apr 2012, *Pingbian Chinese Medicine Expedition 5325230227* (IMDY). Daguan, on the road from Mugan to Sanjiangkou, in the vicinity of 1564 km milestone, 28°10′20″N, 103°58′27″E, 1494 m, 13 Aug 2012, *J. Cai et al. 12CS5458* (KUN); Zhaotong, Qiaojia, 27°00′48″N, 103°14′38″E, 2164 m, 7 Aug 2020, *L. Lu et al. LL-2020-08* (KUN); Dali Prefecture, Cangshan Mountain, 25°48′14″N, 100°06′15″E, 2160 m, 12 Aug 2020, *L. Lu et al. LL-2020-13* (KUN); Baoshan, Tengchong, Houqiao, Danzha Village, 25°29′22″N, 98°14′43″E, 1940 m, 13 Aug 2020, *L. Lu et al. LL-2020-14* (KUN); Baoshan, Tengchong, Yawushang Village, 25°24′42″N, 98°30′05″E, 2080 m, 14 Aug 2020, *L. Lu et al. LL-2020-16* (KUN); Baoshan, Tengchong, Near the Qing Shui Airport, 24°58′26″N, 98°28′30″E, 1625 m, 31 Aug 2011, *Y.Z. Zhou Zhyz-521* (KUN); Baoshan, Tengchong, Dashuijing, Rui Dian Township, 25°26′01″N, 98°28′00″E, 2000 m, 18 Oct 1977, *P.H. Shun 20525* (HITBC); Baoshan, Tengchong, Wenhua Village, Back Mountain of Huapo, 2200 m, 21 Nov 1978, *780 Engineering 0821* (PE); Baoshan, Changning, Goujie Village, 25°02′30″N, 99°50′37″E, 2130 m, 15 Aug 2020, *L. Lu et al. LL-2020-21* (KUN); Wenshan, Guangnan, Mawu Village, 23°41′15″N, 104°51′12″E, 1561 m, 27 Aug 2020, *L. Lu et al. LL-2020-33* (KUN); Wenshan, Malipo, Mali Town, 23°09′55″N, 104°44′28″E, 1370 m, 28 Aug 2020, *L. Lu et al. LL-2020-34* (KUN); Wenshan, Gumu, Tianchong Village, 23°13′12″N, 104°12′01″E, 1595 m, 29 Aug 2020, *L. Lu et al. LL-2020-36* (KUN); Honghe Prefecture, Pingbian, Yuping Town, 22°58′16″N, 103°41′16″E, 1604 m, 30 Aug 2020, *L. Lu et al. LL-2020-37* (KUN); Yuxi, Yuanjiang, Yangchajie Village, 23°39′44″N, 101°45′45″E, 2151 m, 2 Sep 2020, *L. Lu et al. LL-2020-46* (KUN); Yuxi, Hontaqu, Luohe Village, 24°23′03″N, 102°19′13″E, 2224 m, 3 Sep 2020, *L. Lu et al. LL-2020-47* (KUN); Chuxiong Prefecture, Wuding, Gaoqiao Town, 25°39′21″N, 102°05′43″E, 2607 m, 5 Sep 2020, *L. Lu et al. LL-2020-50* (KUN); Qujing, Huize, on the north side of the back hill of Leye Town, 26°31′08″N, 103°27′03″E, 2038 m, 7 Sep 2020, *L. Lu et al. LL-2020-55* (KUN); Kunming, Songming, Xiaozhuyuan Village, 25°21′40″N, 102°58′52″E, 2012 m, 26 Sep 2020, *L. Lu et al. LL-2020-58* (KUN).

**2 *Gaultheria luchunensis*** Yi R. Li, Lu Lu & P.W. Fritsch sp. nov.

**Additional specimens examined: China. Yunnan:** Luchun County. Erpu to Banpo roadside, ca. 1100 m elev., 22 Oct 2000, *Y.M.* *Shui & W.H. Chen 13662* (KUN); Luchun, Erpu to Banpo roadside, ca. 1100 m elev., 22 Oct 2000, *Y.M.* *Shui & W.H. Chen 13704* (KUN); Luchun, Erpu to Banpo roadside, ca. 1100 m elev., 22 Oct 2000, *Y.M.* *Shui & W.H. Chen 14087* (KUN).

**3** ***Gaultheria mangshanensis*** Yi R. Li, Lu Lu & P.W. Fritsch sp. nov.

**Additional specimens examined:** none

**4** ***Gaultheria pingbienensis*** (C.Y. Wu ex T.Z. Xu) Yi R. Li, Lu Lu & P.W. Fritsch comb. & stat. nov.

**Additional specimens examined:** **China. Yunnan:** Pingbian, 1400 m, 1 Jun 1934, *X.T. Cai* *60059* (KUN); Pingbian, Adakou Kozai, 1760 m, 24 Apr 2012, *Pingbian County Chinese Medicine Resources Census Team* *5325230227* (IMDY).

**5 *Gaultheria* *wuliangshanensis*** Yi R. Li, Lu Lu & P.W. Fritsch sp. nov.

**Additional specimens examined:** **China. Yunnan:** Jingdong, Wuliang Mountain, 2800 m, 12 Nov 1956, *B.Y. Qiu* *53567* (KUN); Jingdong, Wuliang Mountain, 2600 m, 15 Nov 1956, *B.Y. Qiu* *53703* (KUN).
