# Supplementary figures and images for: Patterns of genetic variation and morphology support the recognition of five species in the Gaultheria leucocarpa Blume (Ericaceae) group from mainland China
Source: Ecol Evol. 2023 Jun 9;13(6):e10178. doi: 10.1002/ece3.10178 (PMC10251198; doi:10.1002/ece3.10178)

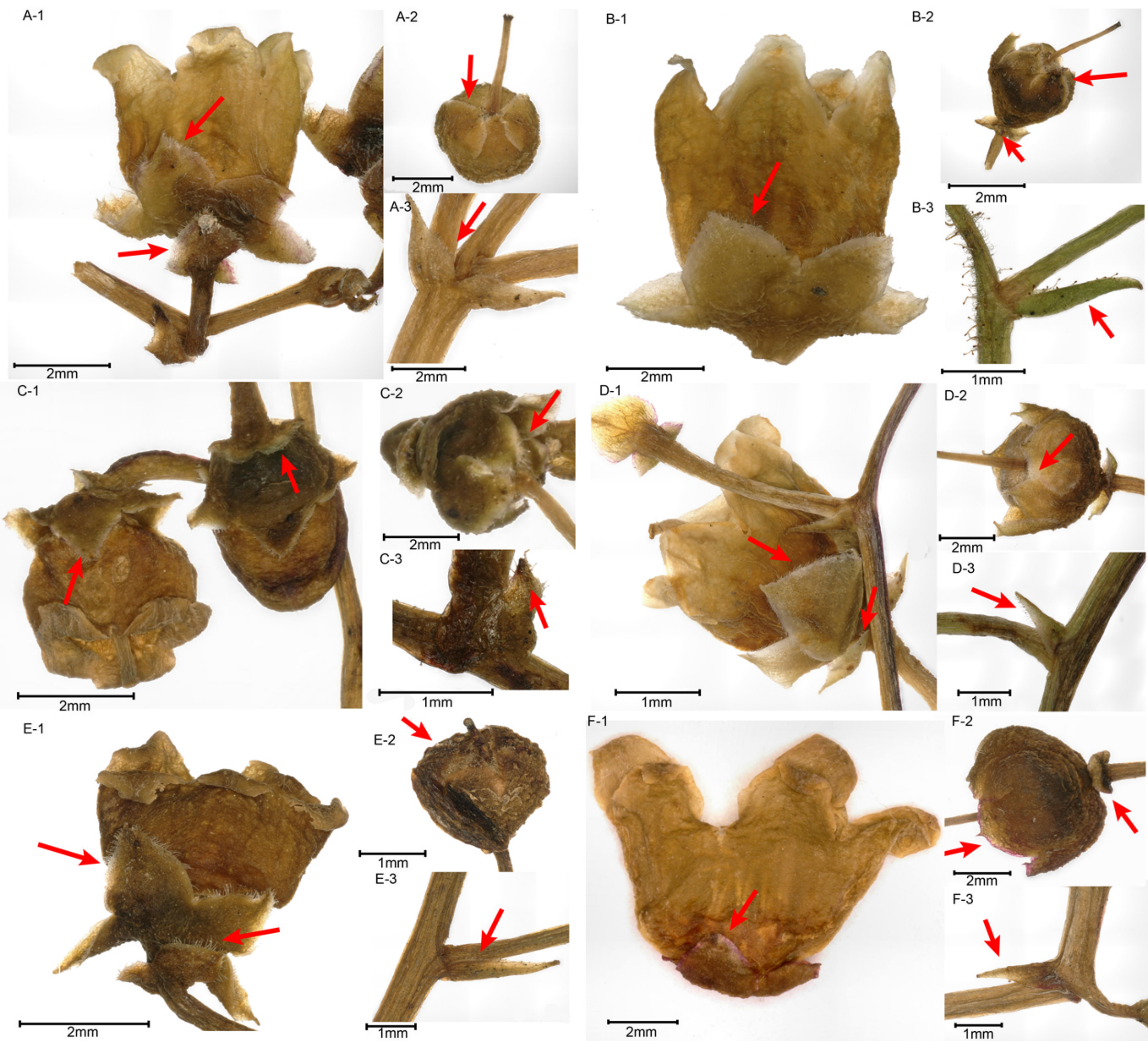

Figure S2

Supplement: Supplementary file 9 — Figure S2 [file ECE3-13-e10178-s003.pdf]
