# Supplementary material for: Patterns of genetic variation and morphology support the recognition of five species in the Gaultheria leucocarpa Blume (Ericaceae) group from mainland China
Source: Ecol Evol. 2023 Jun 9;13(6):e10178. doi: 10.1002/ece3.10178 (PMC10251198; doi:10.1002/ece3.10178)
Supplement: Supplementary file 10 — Tables S1–S6 [file ECE3-13-e10178-s009.docx]

**Table S1** Current taxonomy of *Gaultheria leucocarpa* outside mainland China (Sleumer, 1967)

| Taxon | Original publication | Distribution | Diagnostic characters |
| --- | --- | --- | --- |
| *G. leucocarpa* Bl. var. *leucocarpa* | | | |
| *G. leucocarpa* var. *leucocarpa* f. *leucocarpa* | Bijdr. Fl. Ned. Ind. 15: 856. 1826 | Java and Sumatra (rare) | Inflorescence glabrous, ovary densely pubescent, fruit white or rose-tinged |
| *G. leucocarpa* var. *leucocarpa* f. *scandens* Hochr. | Candollea 2: 494. 1925 | Java and Sumatra (rare) | Inflorescence densely yellowish short-pubescent, ovary densely pubescent, fruit white or with pink hue |
| *G. leucocarpa* var. *leucocarpa* f. *cumingiana* (Vidal) Sleumer | Reinwardtia 4: 179. 1957 | Taiwan, Upper Myanmar, Indochina, Thailand, Peninsular Malaysia, Sumatra, Java, and the Philippines | Inflorescence glabrous, ovary densely pubescent, fruit dark red to purplish black |
| *G. leucocarpa* var. *leucocarpa* f. *melanocarpa* J. J. Sm. ex Amshoff | Bekn. Fl. Java 7: 5. 1948 | Malay Peninsula, Sumatra, and Java | Inflorescence densely yellowish short-pubescent, ovary densely pubescent, fruit dark red to purplish blackish |
| *G. leucocarpa* var. *hirta* Valeton ex J. J. Sm. | Bull. Jard. Bot. Buitenzorg III, 13: 454. 1935 | Tapanuli, North Sumatra | Branchlets, leaves beneath, inflorescence, and corolla densely glandular-setose, patent-pubescent, or pubescent |
| *G. leucocarpa* var. *psilocarpa* (H. F. Copel.) Sleumer | Reinwardtia 4: 183. 1957 | Taiwan of China and the Philippines | Inflorescence glabrous, ovary glabrous, fruit dark red to purplish blackish |

**Table S2** Voucher information of populations of the *Gaultheria leucocarpa* group from mainland China sampled in this study.

| Population code | Collection information | Locality | Latitude (N) | Longitude (E) | Elevation (m) |
| --- | --- | --- | --- | --- | --- |
| *Gaultheria leucocarpa* var. *crenulata* | | | | | |
| WD | L. Lu et al., LL-2020-50 | Guanpo, Wuding, Yunnan, China | 25°39′ | 102°05′ | 2607 |
| *Gaultheria leucocarpa* var. *leucocarpa* f. *cumingiana* | | | | | |
| F | D.S. Penneys 2355 | Mindanao, Davao del Sur, Philippines | 6°59' | 125°16′ | 2825 |
| *Gaultheria leucocarpa* var. *leucocarpa* f. *leucocarpa* | | | | | |
| LYW | K.M. Wong | Ulu Kali Mountain, Selangor-Pahang, Malaysia | 3°25′ | 101°47′ | 1748 |
| *Gaultheria leucocarpa* var. *pingbienensis* | | | | | |
| DWS | L. Lu et al., LL-2020-39 | Dawei Mountain, Pingbian, Yunnan, China | 22°55′ | 103°41′ | 2074 |
| *Gaultheria leucocarpa* var. *yunnanensis* | | | | | |
| DHY | L. Lu et al., LL-2019-52 | Qutan, Heyuan, Guangdong, China | 24°28′ | 114°44' | 935 |
| DL | L. Lu et al., LL-2020-13 | Cang Mountain, Dali, Yunnan, China | 25°48′ | 100°06′ | 2179 |
| HXQ | X.Q. He HXQ-1001 | Yu Mountain, Taiwan, China | 23°27′ | 120°54′ | 1774 |
| JWS | L. Lu et al., LL-2020-28 | Jiuwan Mountain, Liuzhou, Guangxi, China | 25°13′ | 108°40′ | 900 |
| PB | L. Lu et al., LL-2020-37 | Township, Pingbian, Yunnan, China | 22°53′ | 103°42′ | 2239 |
| SGL | L. Lu et al., LL-2020-05 | Lu Mountain, Xichang, Sichuan, China | 27°49′ | 101°47′ | 1702 |
| YGN | L. Lu et al., LL-2020-33 | Mawu, Guangnan, Yunnan, China | 23°41′ | 104°51′ | 1561 |
| Unusual populations | | | | | |
| JD | L. Lu et al., LL-2020-25 | Wuliang Mountain, Jingdong, Yunnan, China | 24°21′ | 100°44′ | 2300 |
| MS | L. Lu et al., LL-2019-36 | Mang Mountain, Yizhang, Hunan, China | 24°57′ | 112°57′ | 1222 |
| YLC | L. Lu et al., LL-2020-44 | Fenshuiling, Luchun, Yunnan, China | 22°59′ | 102°27′ | 2026 |

**Table S3** The genic regions and primer sequences employed for this study.

| **Region** | **Primer name** | **Primer sequence (5'to 3')** | **References** |
| --- | --- | --- | --- |
| *rpl16* | *rpl*16-F71 | GCTATGCTTAGTGTGTGACTCGTTG | Lu et al., 2010. Molecular Phylogenetics and Evolution, 57: 364–379. |
|  | *rpl*16-R1516 | CCCTTCATTCTTCCTCTATGTTG |  |
| *matK* | *matK*1F | ATGGAGGAATTCAAAAGAAATTTAG |  |
|  | *matK*1100R | CGTGCT TGCATT TTTCATTGC |  |
| *trnL-trnF* | *trnL* | CGAAATCGGTAGACGCTACG |  |
|  | *trnF* | ATTTGAACTGGTGACACGAG |  |
| ITS | ITS-4 | TCCTCCGCTTATTGATATGC |  |
|  | ITS-5 | GGAAGGAGAAGTCGTAACAAGG |  |
| *rpl33-psaJ* | *rpl33-*F | TCCGGATGCGTTAACATTCCCCTT | Li et al., 2020. Guihaia, 40(1): 71–82. |
|  | *psaJ*-R | CCTTGGAAGGGTAACACACAGGTGC |  |
| *rpl32-trnL* | *trnL* | CTGCTTCCTAAGAGCAGCGT |  |
|  | *rpL32* | CAGTTCCAAAAAAACGTACTTC |  |
| *AAT* | F253 | CACAGCTGGAAGCACATCAC | Gong & Gong, 2016. Tree Genetics & Genomes, 12(5): 1–15. |
|  | R581 | GCACTTTTAGCATGGAAGGC |  |
| *LOC* | *LOC*-F | AGTAATTGTGGGCTTGGATT | This study |
|  | *LOC*-R | TAAGGGTAACACCTTGCATCT |  |

**Table S4** List of vouchers and GenBank access numbers for taxa downloaded from GenBank for use in the phylogenetic analysis of *Gaultheria.*

| Species name | *matK* | *rpl16* | *trnL-trnF* | ITS |
| --- | --- | --- | --- | --- |
| *Eubotrys racemosa* Nutt. | JF801301 | JF801459 | JF801632 | JF801556 |
| *Eubotrys recurva* (Buckley) Britton | JF801302 | JF801460 | JF801633 | JF801557 |
| *Gaultheria adenothrix* (Miq.) Maxim. | JF801345 | JF801506 | JF801679 | FJ010595 |
| *Gaultheria amoena* A.C. Sm. | AF366626 | JF801490 | JF801663 | JF801582 |
| *Gaultheria antarctica* Hook. f. | JF801380 | JF801547 | JF801720 | JF801618 |
| *Gaultheria appressa* A.W. Hill | JF801366 | JF801533 | JF801706 | FJ665700 |
| *Gaultheria appressa* A.W. Hill | FJ665745 | JF801532 | JF801705 | FJ665701 |
| *Gaultheria beccarii* Kron & P.W. Fritsch | JF801307 | JF801468 | JF801641 | JF801564 |
| *Gaultheria buxifolia* Willd. | JF801359 | JF801523 | JF801696 | JF801609 |
| *Gaultheria caespitosa* Poepp. & Endl. | JF801381 | JF801548 | JF801721 | JF801619 |
| *Gaultheria cardiosepala* Hand.-Mazz. | HM597394 | HM597481 | HM597568 | HM597307 |
| *Gaultheria chrysothrix* (Stapf) Kron & P.W. Fritsch | JF801317 | JF801478 | JF801651 | JF801572 |
| *Gaultheria ciliolata* (Hook. f.) F. Muell. | JF801314 | JF801475 | JF801648 | JF801570 |
| *Gaultheria cinnamomifolia* (Stapf) Kron & P.W. Fritsch | JF801324 | JF801484 | JF801657 | JF801576 |
| *Gaultheria cuneata* (Rehder & E.H. Wilson) Bean | HM597337 | HM597424 | HM597511 | HM597250 |
| *Gaultheria discolor* Nutt. ex Hook. | HM597366 | HM597453 | HM597540 | HM597279 |
| *Gaultheria dolichopoda* Airy Shaw | HM597405 | HM597492 | HM597579 | HM597318 |
| *Gaultheria domingensis* Urb. | JF801342 | JF801503 | JF801676 | JF801594 |
| *Gaultheria dumicola* W.W. Sm. | JF801382 | JF801549 | JF801722 | JF801620 |
| *Gaultheria erecta* Vent. | JF801332 | JF801493 | JF801666 | JF801585 |
| *Gaultheria eriophylla* (Pers.) Mart. ex Sleumer | JF801337 | JF801498 | JF801671 | JF801589 |
| *Gaultheria foliolosa* Benth. | AF366632 | JF801524 | JF801697 | JF801610 |
| *Gaultheria fragrantissima* Wall. | JF801384 | JF801552 | JF801725 | JF801623 |
| *Gaultheria glomerata* (Cav.) Sleumer | JF801340 | JF801501 | JF801674 | JF801592 |
| *Gaultheria griffithiana* Wight | HM597355 | HM597442 | HM597529 | HM597268 |
| *Gaultheria hispida* R.Br. | JF801367 | JF801534 | JF801707 | FJ665707 |
| *Gaultheria hispidula* (L.) Muhl. ex Bigelow | AF366634 | JF801465 | JF801638 | JF801562 |
| *Gaultheria hookeri* C.B. Clarke | HM597360 | HM597447 | HM597534 | HM597273 |
| *Gaultheria humifusa* (Graham) Rydb. | JF801346 | JF801507 | JF801680 | FJ665708 |
| *Gaultheria hypochlora* Airy Shaw | HM597422 | HM597506 | HM597593 | HM597332 |
| *Gaultheria insana* (Molina) D.J. Middleton | JF801354 | JF801517 | JF801690 | JF801604 |
| *Gaultheria itatiaiae* Wawra | JF801360 | JF801527 | JF801700 | JF801611 |
| *Gaultheria lanceolata* Hook. f. | JF801368 | JF801535 | JF801708 | FJ665710 |
| *Gaultheria luzonica* A. Gray | JF801321 | JF801482 | JF801655 | FJ010600 |
| *Gaultheria macrostigma* (Colenso) D.J. Middleton | JF801369 | JF801536 | JF801709 | FJ665711 |
| *Gaultheria malayana* Airy Shaw | HM597367 | HM597454 | HM597541 | HM597280 |
| *Gaultheria memecyloides* (Stapf) Kron & P.W. Fritsch | JF801310 | JF801471 | JF801644 | JF801567 |
| *Gaultheria mucronata* (L. f.) Hook. & Arn. | FJ010622 | JF801513 | JF801686 | FJ010604 |
| *Gaultheria myrsinoides* Kunth | JF801352 | JF801515 | JF801688 | JF801602 |
| *Gaultheria myrtilloides* Cham. & Schltdl. | JF801361 | JF801528 | JF801701 | JF801612 |
| *Gaultheria nubigena* (Phil.) B.L. Burtt & Sleumer | FJ665808 | JF801512 | JF801685 | FJ665712 |
| *Gaultheria ovatifolia* A. Gray | JF801347 | JF801508 | JF801681 | JF801597 |
| *Gaultheria penduliflora* (Stapf) Kron & P.W. Fritsch | JF801319 | JF801480 | JF801653 | JF801573 |
| *Gaultheria phillyreifolia* (Pers.) Sleumer | JF801355 | JF801518 | JF801691 | JF801605 |
| *Gaultheria pinifolia* (Stapf) Kron & P.W. Fritsch | JF801313 | JF801474 | JF801647 | JF801569 |
| *Gaultheria poeppigii* DC. | JF801351 | JF801514 | JF801687 | JF801601 |
| *Gaultheria praticola* C.Y. Wu | JF801383 | JF801551 | JF801724 | JF801622 |
| *Gaultheria prostrata* W.W. Sm. | JF801348 | JF801510 | JF801683 | JF801599 |
| *Gaultheria pumila* (L. f.) D.J. Middleton | JF801357 | JF801520 | JF801693 | AF358896 |
| *Gaultheria punctulata* (Stapf) Kron & P. W. Fritsch | JF801312 | JF801473 | JF801646 | JF801568 |
| *Gaultheria pyrolifolia* Hook. f. ex C.B. Clarke | HM597380 | HM597467 | HM597554 | HM597293 |
| *Gaultheria pyroloides* Hook. f. & Thomson ex Miq. | JF801349 | JF801511 | JF801684 | HM597252 |
| *Gaultheria reticulata* Kunth | JF801330 | JF801491 | JF801664 | JF801583 |
| *Gaultheria rupestris* (L. f.) D. Don. | JF801379 | JF801546 | JF801719 | FJ665716 |
| *Gaultheria sanguinolenta (Sleumer)* Kron & P.W. Fritsch | JF801327 | JF801487 | JF801660 | JF801579 |
| *Gaultheria schultesii* Camp | JF801334 | JF801495 | JF801668 | FJ010601 |
| *Gaultheria semi-infera* (C.B. Clarke) Airy Shaw | HM597386 | HM597473 | HM597560 | HM597299 |
| *Gaultheria shallon* Pursh | JF801329 | JF801489 | JF801662 | JF801581 |
| *Gaultheria sphenophylla* (Sleumer) Kron & P.W. Fritsch | JF801309 | JF801470 | JF801643 | JF801566 |
| *Gaultheria straminea* R.C. Fang | HM597390 | HM597476 | HM597563 | HM597302 |
| *Gaultheria suborbicularis* W.W. Sm. | JF801305 | JF801466 | JF801639 | JF801205 |
| *Gaultheria tenuifolia* (Phil.) Sleumer | JF801356 | JF801519 | JF801692 | JF801606 |
| *Gaultheria thymifolia* Stapf ex Airy Shaw | HM597396 | HM597483 | HM597570 | HM597309 |
| *Gaultheria tomentosa* Kunth | JF801339 | JF801500 | JF801673 | JF801591 |
| *Gaultheria trichophylla* Royle | HM597413 | HM597496 | HM597583 | HM597322 |

**Table S5** GenBank accession numbers for populations and taxa of the *Gaultheria leucocarpa* group newly obtained for this study*.*

| Samples (Population code & individual number) | Taxon name prior to revision | Taxon name after revision | *matK* | *rpl16* | *trnL-trnF* | ITS |
| --- | --- | --- | --- | --- | --- | --- |
| WD14 | *G. leucocarpa* var. *crenulata* | *G. crenulata* | OL555749 | OL555773 | OL661374 | OL583973 |
| WD15 | *G. leucocarpa* var. *crenulata* | *G. crenulata* | OL555750 | OL555774 | OL661375 | OL583974 |
| F32 | *G. leucocarpa* var. *leucocarpa* f. *cumingiana* | [inapplicable](javascript:;) | OM048990 | OM032568 | OM032574 | OM022924 |
| F34 | *G. leucocarpa* var. *leucocarpa* f. *cumingiana* | [inapplicable](javascript:;) | OM048991 | OM032569 | OM032575 | OM022925 |
| LYW5 | *G. leucocarpa* var. *leucocarpa* f. *leucocarpa* | [inapplicable](javascript:;) | OM048994 | OM032572 | OM032578 | OM022928 |
| LYW6 | *G. leucocarpa* var. *leucocarpa* f. *leucocarpa* | [inapplicable](javascript:;) | OM048995 | OM032573 | OM032579 | OM022929 |
| DWS2 | *G. leucocarpa* var. *pingbienensis* | *G. pingbienensis* | OL555731 | OL555755 | OL661356 | OL583955 |
| DWS4 | *G. leucocarpa* var. *pingbienensis* | *G. pingbienensis* | OL555732 | OL555756 | OL661357 | OL583956 |
| DHY5 | *G. leucocarpa* var. *yunnanensis* | *G. crenulata* | OL555739 | OL555763 | OL661364 | OL583963 |
| DHY6 | *G. leucocarpa* var. *yunnanensis* | *G. crenulata* | OL555740 | OL555764 | OL661365 | OL583964 |
| DL1 | *G. leucocarpa* var. *yunnanensis* | *G. crenulata* | OL555741 | OL555765 | OL661366 | OL583965 |
| DL3 | *G. leucocarpa* var. *yunnanensis* | *G. crenulata* | OL555742 | OL555766 | OL661367 | OL583966 |
| HXQ3 | *G. leucocarpa* var. *yunnanensis* | [inapplicable](javascript:;) | OM048992 | OM032570 | OM032576 | OM022926 |
| HXQ4 | *G. leucocarpa* var. *yunnanensis* | [inapplicable](javascript:;) | OM048993 | OM032571 | OM032577 | OM022927 |
| JWS1 | *G. leucocarpa* var. *yunnanensis* | *G. crenulata* | OL555743 | OL555767 | OL661368 | OL583967 |
| JWS3 | *G. leucocarpa* var. *yunnanensis* | *G. crenulata* | OL555744 | OL555768 | OL661369 | OL583968 |
| PB1 | *G. leucocarpa* var. *yunnanensis* | *G. crenulata* | OL555745 | OL555769 | OL661370 | OL583969 |
| PB2 | *G. leucocarpa* var. *yunnanensis* | *G. crenulata* | OL555746 | OL555770 | OL661371 | OL583970 |
| SGL1 | *G. leucocarpa* var. *yunnanensis* | *G. crenulata* | OL555747 | OL555771 | OL661372 | OL583971 |
| SGL2 | *G. leucocarpa* var. *yunnanensis* | *G. crenulata* | OL555748 | OL555772 | OL661373 | OL583972 |
| YGN1 | *G. leucocarpa* var. *yunnanensis* | *G. crenulata* | OL555751 | OL555775 | OL661376 | OL583975 |
| YGN2 | *G. leucocarpa* var. *yunnanensis* | *G. crenulata* | OL555752 | OL555776 | OL661377 | OL583976 |
| JD1 | [inapplicable](javascript:;) | *G. wuliangshanensis* | OL555733 | OL555757 | OL661358 | OL583957 |
| JD3 | [inapplicable](javascript:;) | *G. wuliangshanensis* | OL555734 | OL555758 | OL661359 | OL583958 |
| MS13 | [inapplicable](javascript:;) | *G. mangshanensis* | OL555729 | OL555753 | OL661354 | OL583953 |
| MS15 | [inapplicable](javascript:;) | *G. mangshanensis* | OL555730 | OL555754 | OL661355 | OL583954 |
| YLC5 | [inapplicable](javascript:;) | *G. luchunensis* | OL555737 | OL555761 | OL661362 | OL583961 |
| YLC7 | [inapplicable](javascript:;) | *G. luchunensis* | OL555738 | OL555762 | OL661363 | OL583962 |

**Table S6** GenBank access numbers for populations and taxa prior and after revision of the *Gaultheria leucocarpa* group from mainland China newly obtained for the population genetic analysis.

| Samples (Population code & individual number) | Taxon name prior to revision | Species name after revision | *rpl32-trnL* | *rpl33-psaJ* | *AAT* | *LOC* |
| --- | --- | --- | --- | --- | --- | --- |
| WD1 | *G. leucocarpa* var. *crenulata* | *G. crenulata* | OL661388 | OL661498 | OL870644 | OL870754 |
| WD2 | *G. leucocarpa* var. *crenulata* | *G. crenulata* | OL661389 | OL661499 | OL870645 | OL870755 |
| WD3 | *G. leucocarpa* var. *crenulata* | *G. crenulata* | OL661390 | OL661500 | OL870646 | OL870756 |
| WD4 | *G. leucocarpa* var. *crenulata* | *G. crenulata* | OL661391 | OL661501 | OL870647 | OL870757 |
| WD5 | *G. leucocarpa* var. *crenulata* | *G. crenulata* | OL661392 | OL661502 | OL870648 | OL870758 |
| WD6 | *G. leucocarpa* var. *crenulata* | *G. crenulata* | OL661393 | OL661503 | OL870649 | OL870759 |
| WD7 | *G. leucocarpa* var. *crenulata* | *G. crenulata* | OL661394 | OL661504 | OL870650 | OL870760 |
| WD8 | *G. leucocarpa* var. *crenulata* | *G. crenulata* | OL661395 | OL661505 | OL870651 | OL870761 |
| WD9 | *G. leucocarpa* var. *crenulata* | *G. crenulata* | OL661396 | OL661506 | OL870652 | OL870762 |
| WD10 | *G. leucocarpa* var. *crenulata* | *G. crenulata* | OL661397 | OL661507 | OL870653 | OL870763 |
| DWS1 | *G. leucocarpa* var. *pingbienensis* | *G. pingbienensis* | OL661428 | OL661538 | OL870684 | OL870794 |
| DWS2 | *G. leucocarpa* var. *pingbienensis* | *G. pingbienensis* | OL661429 | OL661539 | OL870685 | OL870795 |
| DWS3 | *G. leucocarpa* var. *pingbienensis* | *G. pingbienensis* | OL661430 | OL661540 | OL870686 | OL870796 |
| DWS4 | *G. leucocarpa* var. *pingbienensis* | *G. pingbienensis* | OL661431 | OL661541 | OL870687 | OL870797 |
| DWS5 | *G. leucocarpa* var. *pingbienensis* | *G. pingbienensis* | OL661432 | OL661542 | OL870688 | OL870798 |
| DWS6 | *G. leucocarpa* var. *pingbienensis* | *G. pingbienensis* | OL661433 | OL661543 | OL870689 | OL870799 |
| DWS7 | *G. leucocarpa* var. *pingbienensis* | *G. pingbienensis* | OL661434 | OL661544 | OL870690 | OL870800 |
| DWS8 | *G. leucocarpa* var. *pingbienensis* | *G. pingbienensis* | OL661435 | OL661545 | OL870691 | OL870801 |
| DWS9 | *G. leucocarpa* var. *pingbienensis* | *G. pingbienensis* | OL661436 | OL661546 | OL870692 | OL870802 |
| DWS10 | *G. leucocarpa* var. *pingbienensis* | *G. pingbienensis* | OL661437 | OL661547 | OL870693 | OL870803 |
| DHY1 | *G. leucocarpa* var. *yunnanensis* | *G. crenulata* | OL661468 | OL661578 | OL870724 | OL870834 |
| DHY2 | *G. leucocarpa* var. *yunnanensis* | *G. crenulata* | OL661469 | OL661579 | OL870725 | OL870835 |
| DHY3 | *G. leucocarpa* var. *yunnanensis* | *G. crenulata* | OL661470 | OL661580 | OL870726 | OL870836 |
| DHY4 | *G. leucocarpa* var. *yunnanensis* | *G. crenulata* | OL661471 | OL661581 | OL870727 | OL870837 |
| DHY5 | *G. leucocarpa* var. *yunnanensis* | *G. crenulata* | OL661472 | OL661582 | OL870728 | OL870838 |
| DHY6 | *G. leucocarpa* var. *yunnanensis* | *G. crenulata* | OL661473 | OL661583 | OL870729 | OL870839 |
| DHY7 | *G. leucocarpa* var. *yunnanensis* | *G. crenulata* | OL661474 | OL661584 | OL870730 | OL870840 |
| DHY8 | *G. leucocarpa* var. *yunnanensis* | *G. crenulata* | OL661475 | OL661585 | OL870731 | OL870841 |
| DHY9 | *G. leucocarpa* var. *yunnanensis* | *G. crenulata* | OL661476 | OL661586 | OL870732 | OL870842 |
| DHY10 | *G. leucocarpa* var. *yunnanensis* | *G. crenulata* | OL661477 | OL661587 | OL870733 | OL870843 |
| DL1 | *G. leucocarpa* var. *yunnanensis* | *G. crenulata* | OL661398 | OL661508 | OL870654 | OL870764 |
| DL2 | *G. leucocarpa* var. *yunnanensis* | *G. crenulata* | OL661399 | OL661509 | OL870655 | OL870765 |
| DL3 | *G. leucocarpa* var. *yunnanensis* | *G. crenulata* | OL661400 | OL661510 | OL870656 | OL870766 |
| DL4 | *G. leucocarpa* var. *yunnanensis* | *G. crenulata* | OL661401 | OL661511 | OL870657 | OL870767 |
| DL5 | *G. leucocarpa* var. *yunnanensis* | *G. crenulata* | OL661402 | OL661512 | OL870658 | OL870768 |
| DL6 | *G. leucocarpa* var. *yunnanensis* | *G. crenulata* | OL661403 | OL661513 | OL870659 | OL870769 |
| DL7 | *G. leucocarpa* var. *yunnanensis* | *G. crenulata* | OL661404 | OL661514 | OL870660 | OL870770 |
| DL8 | *G. leucocarpa* var. *yunnanensis* | *G. crenulata* | OL661405 | OL661515 | OL870661 | OL870771 |
| DL9 | *G. leucocarpa* var. *yunnanensis* | *G. crenulata* | OL661406 | OL661516 | OL870662 | OL870772 |
| DL10 | *G. leucocarpa* var. *yunnanensis* | *G. crenulata* | OL661407 | OL661517 | OL870663 | OL870773 |
| JWS1 | *G. leucocarpa* var. *yunnanensis* | *G. crenulata* | OL661458 | OL661568 | OL870714 | OL870824 |
| JWS2 | *G. leucocarpa* var. *yunnanensis* | *G. crenulata* | OL661459 | OL661569 | OL870715 | OL870825 |
| JWS3 | *G. leucocarpa* var. *yunnanensis* | *G. crenulata* | OL661460 | OL661570 | OL870716 | OL870826 |
| JWS4 | *G. leucocarpa* var. *yunnanensis* | *G. crenulata* | OL661461 | OL661571 | OL870717 | OL870827 |
| JWS5 | *G. leucocarpa* var. *yunnanensis* | *G. crenulata* | OL661462 | OL661572 | OL870718 | OL870828 |
| JWS6 | *G. leucocarpa* var. *yunnanensis* | *G. crenulata* | OL661463 | OL661573 | OL870719 | OL870829 |
| JWS7 | *G. leucocarpa* var. *yunnanensis* | *G. crenulata* | OL661464 | OL661574 | OL870720 | OL870830 |
| JWS8 | *G. leucocarpa* var. *yunnanensis* | *G. crenulata* | OL661465 | OL661575 | OL870721 | OL870831 |
| JWS9 | *G. leucocarpa* var. *yunnanensis* | *G. crenulata* | OL661466 | OL661576 | OL870722 | OL870832 |
| JWS10 | *G. leucocarpa* var. *yunnanensis* | *G. crenulata* | OL661467 | OL661577 | OL870723 | OL870833 |
| PB1 | *G. leucocarpa* var. *yunnanensis* | *G. crenulata* | OL661438 | OL661548 | OL870694 | OL870804 |
| PB2 | *G. leucocarpa* var. *yunnanensis* | *G. crenulata* | OL661439 | OL661549 | OL870695 | OL870805 |
| PB3 | *G. leucocarpa* var. *yunnanensis* | *G. crenulata* | OL661440 | OL661550 | OL870696 | OL870806 |
| PB4 | *G. leucocarpa* var. *yunnanensis* | *G. crenulata* | OL661441 | OL661551 | OL870697 | OL870807 |
| PB5 | *G. leucocarpa* var. *yunnanensis* | *G. crenulata* | OL661442 | OL661552 | OL870698 | OL870808 |
| PB6 | *G. leucocarpa* var. *yunnanensis* | *G. crenulata* | OL661443 | OL661553 | OL870699 | OL870809 |
| PB7 | *G. leucocarpa* var. *yunnanensis* | *G. crenulata* | OL661444 | OL661554 | OL870700 | OL870810 |
| PB8 | *G. leucocarpa* var. *yunnanensis* | *G. crenulata* | OL661445 | OL661555 | OL870701 | OL870811 |
| PB9 | *G. leucocarpa* var. *yunnanensis* | *G. crenulata* | OL661446 | OL661556 | OL870702 | OL870812 |
| PB10 | *G. leucocarpa* var. *yunnanensis* | *G. crenulata* | OL661447 | OL661557 | OL870703 | OL870813 |
| SGL1 | *G. leucocarpa* var. *yunnanensis* | *G. crenulata* | OL661378 | OL661488 | OL870634 | OL870744 |
| SGL2 | *G. leucocarpa* var. *yunnanensis* | *G. crenulata* | OL661379 | OL661489 | OL870635 | OL870745 |
| SGL3 | *G. leucocarpa* var. *yunnanensis* | *G. crenulata* | OL661380 | OL661490 | OL870636 | OL870746 |
| SGL4 | *G. leucocarpa* var. *yunnanensis* | *G. crenulata* | OL661381 | OL661491 | OL870637 | OL870747 |
| SGL5 | *G. leucocarpa* var. *yunnanensis* | *G. crenulata* | OL661382 | OL661492 | OL870638 | OL870748 |
| SGL6 | *G. leucocarpa* var. *yunnanensis* | *G. crenulata* | OL661383 | OL661493 | OL870639 | OL870749 |
| SGL7 | *G. leucocarpa* var. *yunnanensis* | *G. crenulata* | OL661384 | OL661494 | OL870640 | OL870750 |
| SGL8 | *G. leucocarpa* var. *yunnanensis* | *G. crenulata* | OL661385 | OL661495 | OL870641 | OL870751 |
| SGL9 | *G. leucocarpa* var. *yunnanensis* | *G. crenulata* | OL661386 | OL661496 | OL870642 | OL870752 |
| SGL10 | *G. leucocarpa* var. *yunnanensis* | *G. crenulata* | OL661387 | OL661497 | OL870643 | OL870753 |
| YGN1 | *G. leucocarpa* var. *yunnanensis* | *G. crenulata* | OL661448 | OL661558 | OL870704 | OL870814 |
| YGN2 | *G. leucocarpa* var. *yunnanensis* | *G. crenulata* | OL661449 | OL661559 | OL870705 | OL870815 |
| YGN3 | *G. leucocarpa* var. *yunnanensis* | *G. crenulata* | OL661450 | OL661560 | OL870706 | OL870816 |
| YGN4 | *G. leucocarpa* var. *yunnanensis* | *G. crenulata* | OL661451 | OL661561 | OL870707 | OL870817 |
| YGN5 | *G. leucocarpa* var. *yunnanensis* | *G. crenulata* | OL661452 | OL661562 | OL870708 | OL870818 |
| YGN6 | *G. leucocarpa* var. *yunnanensis* | *G. crenulata* | OL661453 | OL661563 | OL870709 | OL870819 |
| YGN7 | *G. leucocarpa* var. *yunnanensis* | *G. crenulata* | OL661454 | OL661564 | OL870710 | OL870820 |
| YGN8 | *G. leucocarpa* var. *yunnanensis* | *G. crenulata* | OL661455 | OL661565 | OL870711 | OL870821 |
| YGN9 | *G. leucocarpa* var. *yunnanensis* | *G. crenulata* | OL661456 | OL661566 | OL870712 | OL870822 |
| YGN10 | *G. leucocarpa* var. *yunnanensis* | *G. crenulata* | OL661457 | OL661567 | OL870713 | OL870823 |
| JD1 | [inapplicable](javascript:;) | *G. wuliangshanensis* | OL661408 | OL661518 | OL870664 | OL870774 |
| JD2 | [inapplicable](javascript:;) | *G. wuliangshanensis* | OL661409 | OL661519 | OL870665 | OL870775 |
| JD3 | [inapplicable](javascript:;) | *G. wuliangshanensis* | OL661410 | OL661520 | OL870666 | OL870776 |
| JD4 | [inapplicable](javascript:;) | *G. wuliangshanensis* | OL661411 | OL661521 | OL870667 | OL870777 |
| JD5 | [inapplicable](javascript:;) | *G. wuliangshanensis* | OL661412 | OL661522 | OL870668 | OL870778 |
| JD6 | [inapplicable](javascript:;) | *G. wuliangshanensis* | OL661413 | OL661523 | OL870669 | OL870779 |
| JD7 | [inapplicable](javascript:;) | *G. wuliangshanensis* | OL661414 | OL661524 | OL870670 | OL870780 |
| JD8 | [inapplicable](javascript:;) | *G. wuliangshanensis* | OL661415 | OL661525 | OL870671 | OL870781 |
| JD9 | [inapplicable](javascript:;) | *G. wuliangshanensis* | OL661416 | OL661526 | OL870672 | OL870782 |
| JD10 | [inapplicable](javascript:;) | *G. wuliangshanensis* | OL661417 | OL661527 | OL870673 | OL870783 |
| MS1 | [inapplicable](javascript:;) | *G. mangshanensis* | OL661478 | OL661588 | OL870734 | OL870844 |
| MS2 | [inapplicable](javascript:;) | *G. mangshanensis* | OL661479 | OL661589 | OL870735 | OL870845 |
| MS3 | [inapplicable](javascript:;) | *G. mangshanensis* | OL661480 | OL661590 | OL870736 | OL870846 |
| MS4 | [inapplicable](javascript:;) | *G. mangshanensis* | OL661481 | OL661591 | OL870737 | OL870847 |
| MS5 | [inapplicable](javascript:;) | *G. mangshanensis* | OL661482 | OL661592 | OL870738 | OL870848 |
| MS6 | [inapplicable](javascript:;) | *G. mangshanensis* | OL661483 | OL661593 | OL870739 | OL870849 |
| MS7 | [inapplicable](javascript:;) | *G. mangshanensis* | OL661484 | OL661594 | OL870740 | OL870850 |
| MS8 | [inapplicable](javascript:;) | *G. mangshanensis* | OL661485 | OL661595 | OL870741 | OL870851 |
| MS9 | [inapplicable](javascript:;) | *G. mangshanensis* | OL661486 | OL661596 | OL870742 | OL870852 |
| MS10 | [inapplicable](javascript:;) | *G. mangshanensis* | OL661487 | OL661597 | OL870743 | OL870853 |
| YLC1 | [inapplicable](javascript:;) | *G. luchunensis* | OL661418 | OL661528 | OL870674 | OL870784 |
| YLC2 | [inapplicable](javascript:;) | *G. luchunensis* | OL661419 | OL661529 | OL870675 | OL870785 |
| YLC3 | [inapplicable](javascript:;) | *G. luchunensis* | OL661420 | OL661530 | OL870676 | OL870786 |
| YLC4 | [inapplicable](javascript:;) | *G. luchunensis* | OL661421 | OL661531 | OL870677 | OL870787 |
| YLC5 | [inapplicable](javascript:;) | *G. luchunensis* | OL661422 | OL661532 | OL870678 | OL870788 |
| YLC6 | [inapplicable](javascript:;) | *G. luchunensis* | OL661423 | OL661533 | OL870679 | OL870789 |
| YLC7 | [inapplicable](javascript:;) | *G. luchunensis* | OL661424 | OL661534 | OL870680 | OL870790 |
| YLC8 | [inapplicable](javascript:;) | *G. luchunensis* | OL661425 | OL661535 | OL870681 | OL870791 |
| YLC9 | [inapplicable](javascript:;) | *G. luchunensis* | OL661426 | OL661536 | OL870682 | OL870792 |
| YLC10 | [inapplicable](javascript:;) | *G. luchunensis* | OL661427 | OL661537 | OL870683 | OL870793 |
