# Supplementary material for: Patterns of genetic variation and morphology support the recognition of five species in the Gaultheria leucocarpa Blume (Ericaceae) group from mainland China
Source: Ecol Evol. 2023 Jun 9;13(6):e10178. doi: 10.1002/ece3.10178 (PMC10251198; doi:10.1002/ece3.10178)
Supplement: Supplementary file 8 — Figure S1 [file ECE3-13-e10178-s007.pdf]

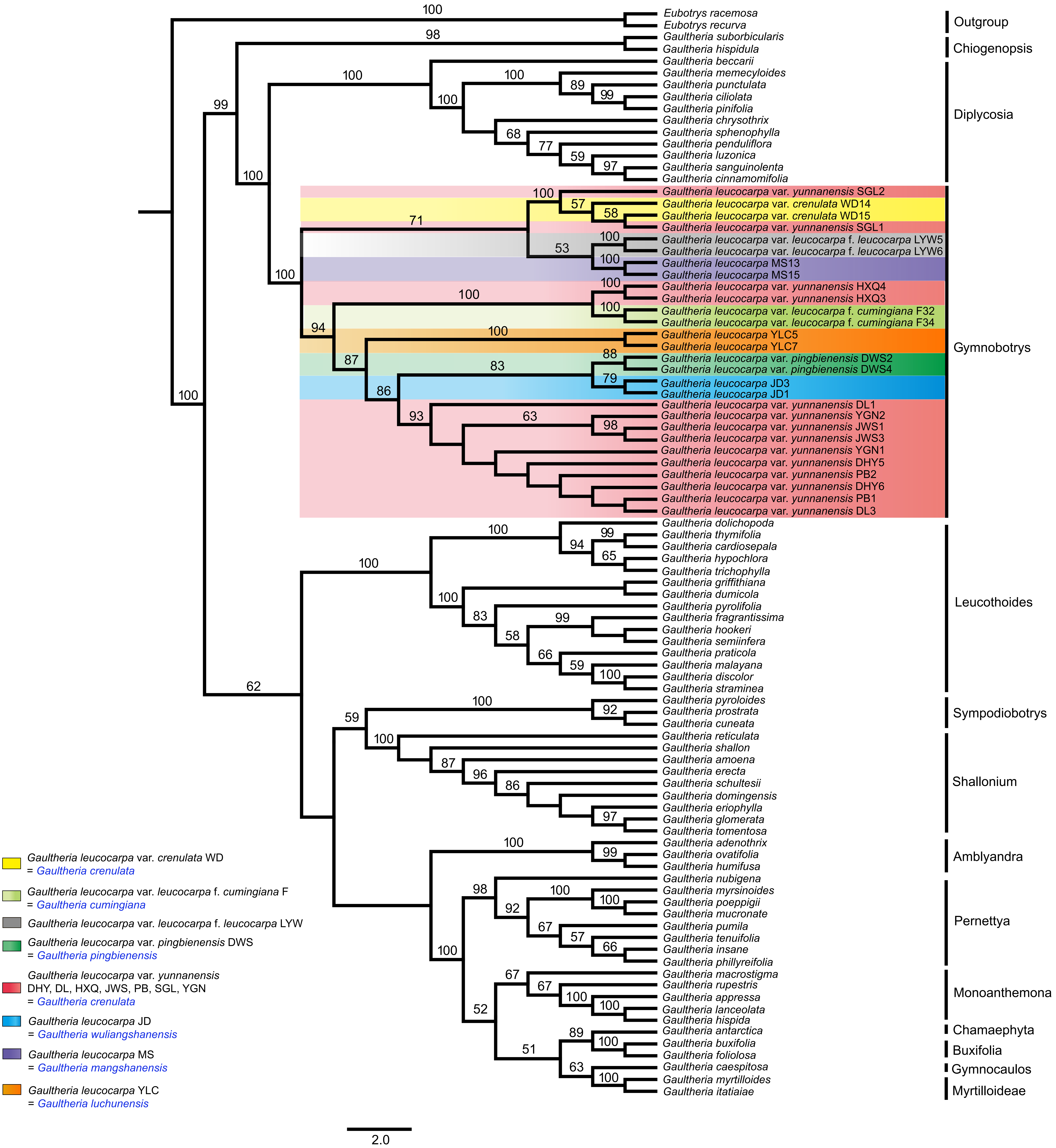

Eubotrys racemosa
Eubotrys recurva
Gaultheria suborbicularis
Gaultheria hispidula
Gaultheria beccarii
Gaultheria memecyloides
Gaultheria punctulata
Gaultheria ciliolata
Gaultheria pinifolia
Gaultheria chrysothrix
Gaultheria sphenophylla
Gaultheria penduliflora
Gaultheria luzonica
Gaultheria sanguinolenta
Gaultheria cinnamomifolia
Gaultheria leucocarpa var. yunnanensis SGL2
Gaultheria leucocarpa var. crenulata WD14
Gaultheria leucocarpa var. crenulata WD15
Gaultheria leucocarpa var. yunnanensis SGL1
Gaultheria leucocarpa var. leucocarpa f. leucocarpa LYW5
Gaultheria leucocarpa var. leucocarpa f. leucocarpa LYW6
Gaultheria leucocarpa MS13
Gaultheria leucocarpa MS15
Gaultheria leucocarpa var. yunnanensis HXQ4
Gaultheria leucocarpa var. yunnanensis HXQ3
Gaultheria leucocarpa var. leucocarpa f. cumingiana F32
Gaultheria leucocarpa var. leucocarpa f. cumingiana F34
Gaultheria leucocarpa YLC5
Gaultheria leucocarpa YLC7
Gaultheria leucocarpa var. pingbienensis DWS2
Gaultheria leucocarpa var. pingbienensis DWS4
Gaultheria leucocarpa JD3
Gaultheria leucocarpa JD1
Gaultheria leucocarpa var. yunnanensis DL1
Gaultheria leucocarpa var. yunnanensis YGN2
Gaultheria leucocarpa var. yunnanensis JWS1
Gaultheria leucocarpa var. yunnanensis JWS3
Gaultheria leucocarpa var. yunnanensis YGN1
Gaultheria leucocarpa var. yunnanensis DHY5
Gaultheria leucocarpa var. yunnanensis PB2
Gaultheria leucocarpa var. yunnanensis DHY6
Gaultheria leucocarpa var. yunnanensis PB1
Gaultheria leucocarpa var. yunnanensis DL3
Gaultheria dolichopoda
Gaultheria thymifolia
Gaultheria cardiosepala
Gaultheria hypochlora
Gaultheria trichophylla
Gaultheria griffithiana
Gaultheria dumicola
Gaultheria pyrolifolia
Gaultheria fragrantissima
Gaultheria hookeri
Gaultheria semiinfera
Gaultheria praticola
Gaultheria malayana
Gaultheria discolor
Gaultheria straminea
Gaultheria pyroloides
Gaultheria prostrata
Gaultheria cuneata
Gaultheria reticulata
Gaultheria shallon
Gaultheria amoena
Gaultheria erecta
Gaultheria schultesii
Gaultheria domingensis
Gaultheria eriophylla
Gaultheria glomerata
Gaultheria tomentosa
Gaultheria adenothrix
Gaultheria ovatifolia
Gaultheria humifusa
Gaultheria nubigena
Gaultheria myrsinoides
Gaultheria poeppigii
Gaultheria mucronate
Gaultheria pumila
Gaultheria tenuifolia
Gaultheria insane
Gaultheria phillyreifolia
Gaultheria macrostigma
Gaultheria rupestris
Gaultheria appressa
Gaultheria lanceolata
Gaultheria hispida
Gaultheria antarctica
Gaultheria buxifolia
Gaultheria foliolosa
Gaultheria caespitosa
Gaultheria myrtilloides
Gaultheria itatiaiae
Outgroup
Chiogenopsis
Diplycosia
Gymnobotrys
Leucothoides
Sympodiobotrys
Shallonium
Amblyandra
Pernettya
Monoanthemona
Chamaephyta
Buxifolia
Gymnocaulos
Myrtilloideae
Gaultheria leucocarpa var. crenulata WD
= Gaultheria crenulata
Gaultheria leucocarpa var. leucocarpa f. cumingiana F
= Gaultheria cumingiana
Gaultheria leucocarpa var. leucocarpa f. leucocarpa LYW
Gaultheria leucocarpa var. pingbienensis DWS
= Gaultheria pingbienensis
Gaultheria leucocarpa var. yunnanensis
DHY, DL, HXQ, JWS, PB, SGL, YGN
= Gaultheria crenulata
Gaultheria leucocarpa JD
= Gaultheria wuliangshanensis
Gaultheria leucocarpa MS
= Gaultheria mangshanensis
Gaultheria leucocarpa YLC
= Gaultheria luchunensis
2.0

Figure S1
